# Standardisation in acute stroke research: A scoping review of upper limb assessments against Stroke Recovery and Rehabilitation Roundtable (SRRR) benchmarks

**DOI:** 10.1177/02692155251398368

**Published:** 2025-11-26

**Authors:** Milica Doric, Lisa Tedesco Triccas, Mingyao Xiong, Faye Tabone, Adrian L Knorz, Nicole Downar, Nick S Ward, Catharina Zich

**Affiliations:** 1 Department of Clinical and Movement Neuroscience, 4919 UCL Queen Square Institute of Neurology, London, UK; 2 Faculty of Rehabilitation Sciences, REVAL Rehabilitation Research Center, Hasselt University, Diepenbeek, Belgium; 3 Wellcome Centre for Integrative Neuroimaging, Nuffield Department of Clinical Neurosciences, 4919 University of Oxford, Oxford, UK; 4 MRC Brain Network Dynamics Unit, Nuffield Department of Clinical Neurosciences, 4919 University of Oxford, Oxford, UK

**Keywords:** Acute stroke, motor assessment, upper limb, scoping review

## Abstract

**Objective:**

To examine how well acute stroke studies assessing upper limb sensorimotor capacity align with the Stroke Recovery and Rehabilitation Roundtable (SRRR) recommendations, focussing on the type of assessment tools used, study and participant characteristics, follow-up timings, and the use of clinical and multimodal data.

**Design:**

Scoping review.

**Data sources:**

Embase, MEDLINE, PubMed, CINAHL, PsycINFO, Google Scholar, and Web of Science were searched for relevant studies published between 01 August 2017 and 30 September 2025.

**Methods:**

This review included studies involving adults with stroke who underwent upper limb assessment during the acute phase. Data were extracted on clinical, structural, and functional assessments, as well as follow-up timing, study, and participant characteristics. Of the 3628 identified articles, 132 met the inclusion criteria.

**Results:**

While global assessments (e.g. NIH stroke scale [NIHSS]) and impairment-level upper limb assessments (e.g. Upper-extremity Fugl-Meyer Assessment) were widely used, activity-level tools (e.g. Action Research Arm Test) were underrepresented. Structural brain imaging was common, though often used only diagnostically, while functional brain imaging and multimodal approaches were rare. Follow-up timing varied, with limited long-term tracking. Demographic reporting was inconsistent, with underrepresentation of young adults and women.

**Conclusion:**

Despite progress, significant gaps remain in the standardisation and comprehensiveness of upper limb assessment in acute stroke research. Future studies should better align with SRRR recommendations to improve data comparability and scientific rigour.

## Clinical messages

The Upper-extremity Fugl-Meyer Assessment (FMA-UE) was the most used upper limb outcome measure in acute stroke, reflecting its clinical utility and established validity in this population.

Activity-based upper limb outcome measures were infrequently used, restricting insights into functional recovery and limiting evaluation across all relevant domains of the International Classification of Functioning, Disability and Health (ICF) framework.

Neuroimaging primarily served diagnostic purposes, with limited application in monitoring recovery or identifying biomarkers for prognosis and personalised rehabilitation planning.

Timing of follow-up assessments was inconsistent, and few studies integrated multimodal data (clinical, kinematic, neuroimaging) as advocated by Stroke Recovery and Rehabilitation Roundtable (SRRR) guidelines.

## Introduction

The global prevalence of stroke exceeds 101 million, with 12.2 million individuals experiencing a first-ever stroke.^1,2^ Stroke remains one of the leading contributors to disability-adjusted life years, with approximately two-thirds of stroke survivors experiencing reduced upper limb sensorimotor capacity.^3,4^ We use upper limb sensorimotor capacity as a unifying descriptor that encompasses the full spectrum of upper limb function, impairment, performance, and recovery, allowing consistent classification across diverse assessment tools and the International Classification of Functioning, Disability and Health (ICF) outcome domains.

Although stroke is a chronic condition, it progresses rapidly during the hyper-acute (0–24 h) and acute (1–7 days) stages.^5^ The acute stage is therefore a critical window for studying upper limb sensorimotor mechanisms, testing therapeutic interventions, and identifying biomarkers of recovery.^6^ However, research in the acute phase faces logistical barriers to patient recruitment.^7^ Thus, despite best efforts, studies are often limited by small sample sizes,^8,9^ significant heterogeneity in stroke pathophysiology,^10^ and inconsistent assessment methodologies^11,12^ – factors that limit generalisation of findings. Greater global harmonisation of terminology, measures, and methods is needed to optimise assessment timing, strengthen meta-analyses, and fuel impactful research. To address these challenges, the SRRR – an international consensus initiative involving researchers, clinicians, methodologists, funders, and people with lived experience – has issued several recommendations.^13^ Key outputs include standardised definitions, consensus frameworks for trial development, and recommended outcome measures for fatigue, balance, mobility, and sensorimotor function. The aim is to accelerate translation of research into practice, reduce study heterogeneity, enhance comparability, and ultimately improve recovery outcomes. For this review, the most relevant SRRR recommendations concern timeline definitions,^5^ biomarkers,^14^ standardised measurement of sensorimotor recovery,^15^ and standardised measurement of quality of movement.^16^

We review the alignment of the research practices in acute stroke upper limb sensorimotor capacity with the SRRR recommendations and identify key gaps. Specifically, this review asks: *To what extent do acute stroke studies assessing upper limb sensorimotor capacity align with the SRRR recommendations?* To address this, we examined: *a) measurement tools used to assess upper limb sensorimotor capacity in the acute phase after stroke; b) study characteristics and participant demographics (e.g. age, sex, time post-stroke); c) follow-up timing.*

## Methods

A focused scoping review, adhering to the proposed Joanna Briggs Institute (JBI) framework,^17^ was conducted to synthesise the literature, identify prevailing trends, and reveal existing gaps in upper limb acute stroke research. We followed the Preferred Reporting Items for Systematic Reviews and Meta-Analyses extension for Scoping Reviews checklist.^18^

## Inclusion criteria

The eligibility criteria for this scoping review were informed by the JBI Population/Concept/Context framework.^19^ *Types of participants:* Adults (18 years and older) who have experienced a stroke and have undergone assessments related to upper limb sensorimotor capacity in the acute stage (1–7 days) post-stroke.^5^ *Concept:* Assessment of upper limb sensorimotor capacity, including global or upper limb specific clinical assessment, and assessment of brain function and brain structure. Assessments may be used for diagnosis, baseline evaluation, or as outcome measure. *Context:* Any healthcare or rehabilitation setting (e.g. hospitals, inpatient rehabilitation units, or acute care facilities), in any geographic location. Only studies published in English were included. *Types of sources of evidence:* Eligible sources included full-text, primary research studies encompassing both quantitative interventional and non-interventional designs. These include randomised and non-randomised controlled trials, pre-post studies, pilot studies, case studies, case–control studies, cross-sectional studies, and cohort studies, allowing for the assessment of broader trends across the field. As the SRRR recommendations are primarily intended for clinical trials, the analyses were repeated with a focus on clinical trials only, where appropriate (Supplementary Results). Conversely, review articles, conference proceedings, protocols, clinical practice guidelines, perspectives, opinion pieces, and evaluations of new devices or measures were excluded, as they were considered inappropriate for addressing the objectives of this scoping review. Grey literature databases were not included in this scoping review.

## Search strategy

Following JBI scoping review methodology, we identified relevant studies through a comprehensive search strategy. We searched (Embase, MEDLINE, PubMed, CINAHL, PsychINFO, Google Scholar, Web of Science) using terms related to (upper limb, motor, acute, and stroke). The full search strategy is provided in Supplementary Table 1. To avoid introducing bias into the search strategy, neither broad assessment categories (i.e. biomarker, electrophysiology, kinematic) nor specific assessments (i.e. Magnetic Resonance Imaging [MRI], Transcranial Magnetic Stimulation [TMS], reaching) were used as search terms for clinical assessments or structural and functional imaging, allowing for the identification of all studies assessing upper limb sensorimotor capacity in acute stroke. The search was conducted on 30 September 2025, and restricted to articles available in English, those containing the specified keywords in the title and/or abstract, and publications between 01 August 2017 and 30 September 2025 (this time frame aligns with the emergence of the SRRR paper on agreed definitions for stroke).^5^

## Source of evidence screening and selection

All retrieved records were entered into EndNote 20 (Clarivate Analytics, PA, USA) referencing software. Duplicate removal was first performed automatically, followed by manual deduplication by MD to ensure accuracy (total number of duplicates = 1421). Screening was conducted in multiple stages: title screening, abstract screening, and comprehensive full-text review. Title and abstract screening were conducted twice by trained independent raters (MD, MX, FT, ND, ALK). Raters were blinded to one another's assessments, and any disagreements were resolved by a third reviewer (CZ) to ensure consistency and adherence to the eligibility criteria. Subsequently, the full-text of the remaining entries was reviewed once. To ensure quality control and reduce intrapersonal variability, one-third of the full-text reviews were independently checked by an additional rater. Reasons for exclusion were documented at the title, abstract, and full-text stage.

## Data extraction

The extraction criteria were established a priori. The following information was extracted: study details (title, author(s), year of publication, study design, country of origin) and sample demographics (sample size, age, gender distribution, time post-stroke). Note that time post-stroke data were standardised and converted into days for consistency (e.g. 1 month = 30 days). Regarding upper limb outcome measures, we focused on clinical assessments spanning motor and global measures of upper limb capacity, as well as measures of brain structure and function. Details on follow-up assessments, including the nature and timing of these evaluations, were also systematically recorded. A complete list of the extracted data elements is provided in the Supplementary Methods. This review does not extract data on study findings, nor does it include any formal assessment of study quality.

## Analysis and presentation of results

The results of the included studies were synthesised using a descriptive, narrative approach, consistent with the objectives of a scoping review. Data were charted and summarised according to key variables, including study characteristics and participant demographics, assessment tools used, and timing of assessments. Findings were organised into figures to support interpretation of patterns, gaps, and trends across the literature. Where appropriate, the analysis was repeated focusing on clinical trials only, as these represent a key area of interest within the SRRR recommendations.

## Results

### Search results

**Figure 1.**
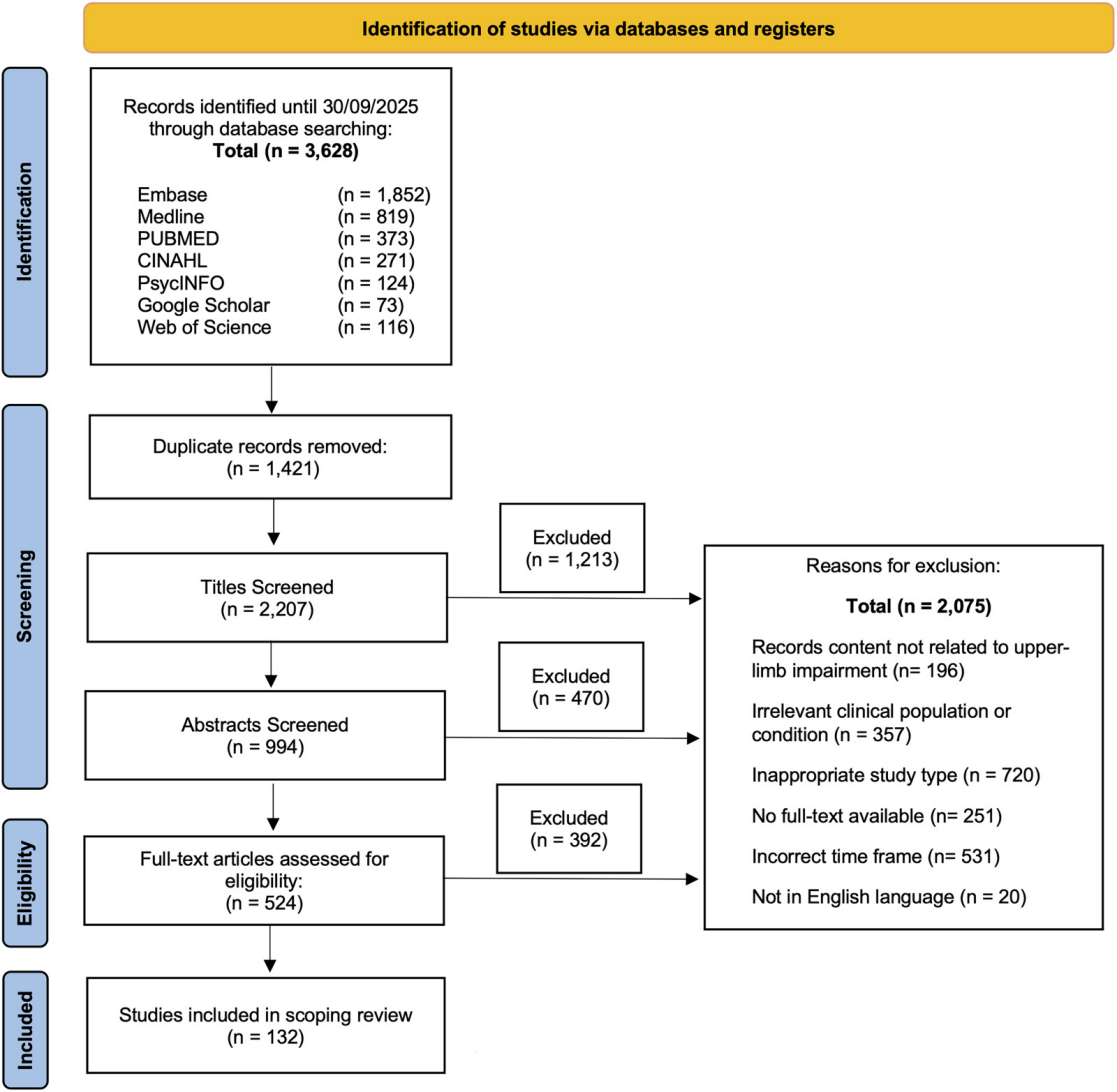
Flow diagram illustrating numbers of articles identified, screened, assessed for eligibility, and included in the review.

The initial search yielded a total of 3628 records, from which 1421 duplicates were removed using EndNote 20 (Clarivate Analytics, PA, USA) and thorough manual verification. Following the application of predefined inclusion and exclusion criteria, an additional 2075 records were excluded during the screening process. This resulted in a final set of 132 original studies deemed eligible for data extraction and synthesis. These publications serve as the foundation of our analysis of upper limb assessments in acute stroke research. The detailed article selection process is outlined in Figure 1. A complete list of the included publications is provided in the Supplementary Methods.

### Discrepancies in the definition of ‘acute’ stroke

Clear definitions are essential for accurate assessment timing in acute stroke research. The widely accepted temporal definition of ‘acute’ stroke refers to 1–7 days post-stroke onset, meaning the assessments evaluating acute upper limb capacity should occur within this timeframe.^5^ However, we identified notable inconsistencies among the included studies. Specifically, 37.9% (*N* = 50) of all studies (41.4% [*N* = 12] of clinical trials) labelled as ‘acute’ conducted initial assessments beyond day 7, effectively diverging into the early subacute phase. Additionally, six studies (two clinical trials) failed to report time post-stroke at their initial upper limb assessments despite categorising their cohorts as acute stroke survivors.

### Study demographics

**Figure 2.**
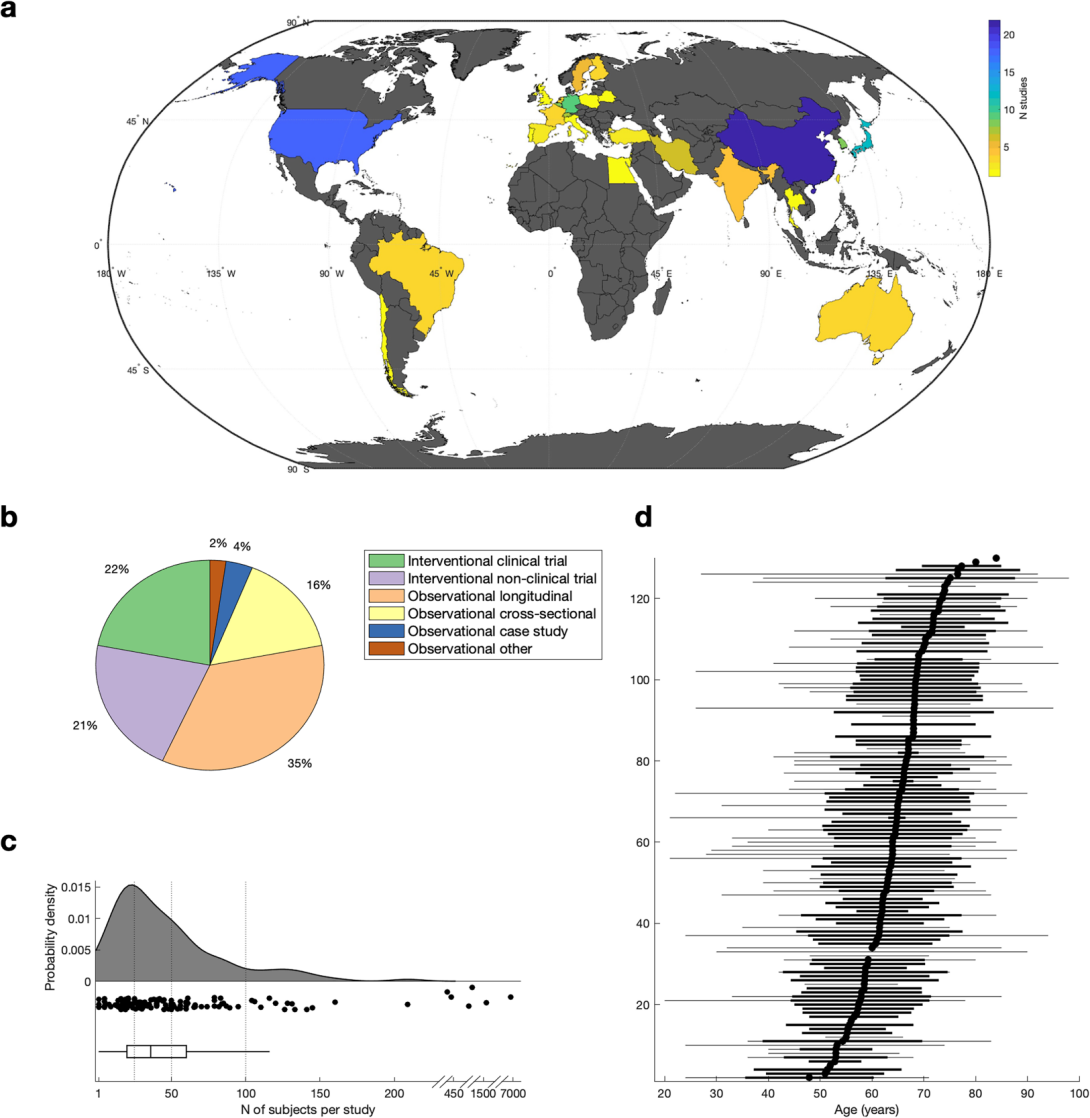
Study demographics. (a) Geographical distribution of included studies. (b) Included studies categorised by study type. (c) Number of subjects per study. Shown is the probability function (top), the corresponding individual datapoints (middle), and the boxplot (bottom). Horizontal line indicates N = 25, N = 50, and N = 100. (d) Age of subjects per study. Each row represents one study in the scoping review, which are sorted based on the average age (i.e. mean or median, depending on data availability). Average age is shown as a block dot, variance (i.e. standard deviation) is shown as thick black line, and age range is shown as thin grey line.

The highest number of publications originated from China (*N* = 22) and the United States of America (*N* = 18, Figure 2(a)). More than half of the studies were observational in design, with 35% (*N* = 46) being longitudinal and 16% (*N* = 21) cross-sectional. Interventional studies accounted for 43% of the studies, whereby 22% (*N* = 29) constitute clinical trials and 21% (*N* = 28) non-clinical trial studies (Figure 2(b)). Sample sizes ranged from 1 to 6801 participants (*M* = 122.0, *Med* = 39.0, *SD* = 604.8, *SE* = 52.6), with 34.8% (*N* = 46) of studies having 25 or fewer subjects (Figure 2(c)). On average more male than female participants were included in these studies (Male: *M* = 70.7, *Med* = 21.0, *SD* = 338.2, *SE* = 7.1; Female: *M* = 53.2, *Med* = 16, *SD* = 273.6, *SE* = 5.7; *t(256)* = 2.99, *p* = 0.003). The mean age across studies was 64.1 years (*Med* = 64.9, *SD* = 8.7, *SE* = 0.2, Figure 2(d)). Among the studies providing an age range (50% [*N* = 66] of the studies) only 28.8% (*N* = 19) have a minimum age below 35 years, illustrating the lack of research in younger stroke survivors (Figure 2(d)). See Supplementary Results for the study demographics of clinical trials only.

### Clinical global assessment after stroke

**Figure 3.**
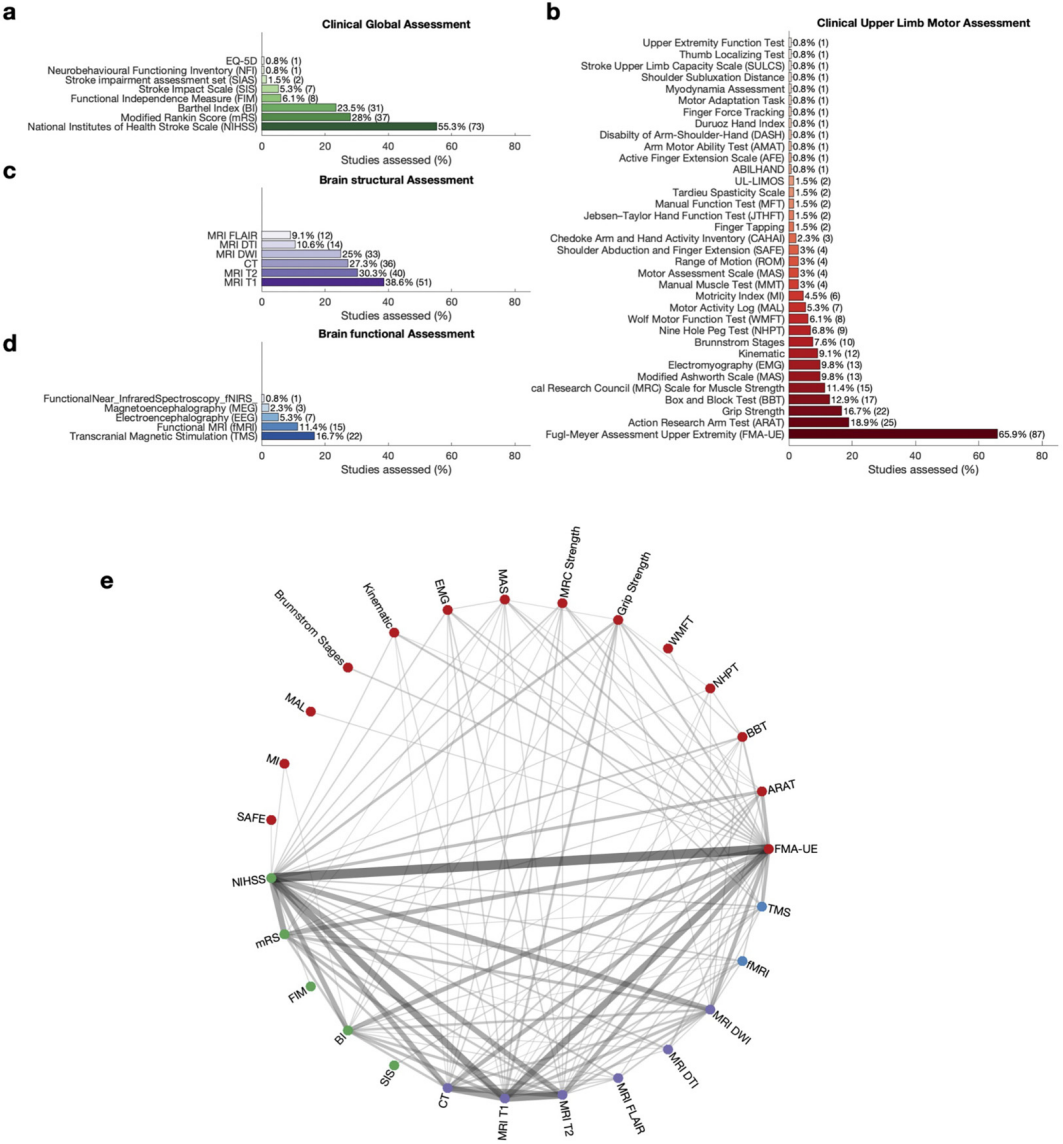
Percentage of assessment categorised by assessment type. (a) Clinical Global Assessment. Assessments are ranked and coloured by the frequency of which they are used. (b) Clinical upper limb Motor Assessment. Assessments are ranked and coloured by the frequency of which they are used. (UL-LIMOS = Upper Limb Lucerne ICF-based Multidisciplinary Observation Scale). (c) Brain structural Assessment. Assessments are ranked and coloured by the frequency of which they are used. (d) Brain functional Assessment. Assessments are ranked and coloured by the frequency of which they are used. (e) Connectivity plot indicating which assessments are obtained together in one study. The colour of each node corresponds to the assessment category; green for Clinical Global Assessment,GCA red for Clinical Upper Limb Motor Assessments, blue for measures of Brain Function, and purple for measures of Brain Structures. Colour and thickness of the edge between two nodes indicates the frequency of which two assessments are used together in one study. Connections that occur less than 3% (N < 5) are omitted from the plot.

At least one clinical measure of global capacity was used in 72.7% (*N* = 96) of all studies (86.2% [*N* = 25] of clinical trials). The NIH stroke scale (NIHSS) was the most frequently employed measure, appearing in 55.3% (*N* = 73) of articles (Figure 3(a), for clinical trials SI Figure 2(a)). This scale is renowned for its robustness in evaluating stroke severity in acute settings.^20,21^ We found that 52 studies used the NIHSS exclusively as a measure to determine stroke severity at baseline, aligning with the SRRR recommendations for routinely early assessment.^15^ Meanwhile, 21 studies used the NIHSS scale as an outcome measure to track changes over time. This dual application highlights the lack of consensus on the most appropriate use of the NIHSS.

The modified Rankin Scale (mRS) was the second most prevalent assessment tool, appearing in 28.0% (*N* = 37) of studies. Additionally, the Barthel Index (BI), which evaluates the ability to perform essential activities of daily living, was employed in 23.5% (*N* = 31) of studies. The prominence of these tools suggests that stroke researchers prioritise both stroke severity and functional independence as key metrics for assessing overall patient outcomes.

Less commonly used measures include the Functional Independence Measure (6.1% [*N* = 8]) and the Stroke Impact Scale (5.3% [*N* = 7]). Additionally, we identified three other global measures, each reported in 2% or fewer (*N* ≤ 2) of the included articles, as illustrated in Figure 3(a). These findings collectively emphasise a preference for established, validated global assessment tools within the field.

### Clinical upper limb motor assessments after stroke

93.9% (*N* = 124) of all studies (100% [*N* = 29] of clinical trials) used at least one clinical measure of upper limb motor assessment. However, the landscape of upper limb motor assessments used was notably heterogeneous (Figure 3(b), for clinical trials SI Figure 2(b)). The Upper-extremity Fugl-Meyer Assessment (FMA-UE) emerged as the predominant tool (65.8% [*N* = 87]). The Action Research Arm Test (ARAT) was the second most common assessment (18.9% [*N* = 25]), followed by Grip Strength (16.7% [*N* = 22]). Beyond these leading assessments, our search uncovered an additional 32 outcome measures, of which 22 were reported in 3% or fewer (*N* ≤ 4) or less of studies. This broad set of tools reflects the complexity and variability of measuring upper limb sensorimotor capacity in acute stroke research.

### Brain structural assessments after stroke

Structural neuroimaging provides a unique window into stroke-related changes in both grey and white matter. Our findings showed that 57.6% (*N* = 76) of all studies (41.7% [*N* = 15] of clinical trials) employed some form of brain structural assessment, with MRI being the most routinely employed technique (Figure 3(c), for clinical trials SI Figure 2(c)). T1-weighted MRI was the most frequently reported imaging modality (38.6%, [*N* = 51]), followed by T2-weight MRI and Computed Tomography (CT) with 30.3% (*N* = 40) and 27.3% (*N* = 36), respectively. Diffusion Weighted Imaging was used in 25.0% (*N* = 33), while Diffusion Tensor Imaging and Fluid-Attenuated Inversion Recovery (FLAIR) occurred in 10.6% (*N* = 14) and 9.1% (*N* = 12) of the studies. Among the studies employing structural neuroimaging, a little under half (42.1% [*N* = 32]) applied it exclusively for stroke diagnosis confirmation, without using it for further outcome evaluation.

### Brain functional assessments after stroke

Measures of brain function offer valuable insights into stroke-related changes in brain function (e.g. functional connectivity, cortical excitability). However, our findings indicate a comparatively lower use of functional assessments, with only 31.1% (*N* = 41) of all studies (20.7% [*N* = 6] of clinical trials). TMS was the most often used technique, seen in 16.7% (*N* = 22) of articles (Figure 3(d), for clinical trials SI Figure 2(d)), with Motor Evoked Potentials (15.9% [*N* = 21]) and Motor Threshold (11.3% [*N* = 15]) being most frequently assessed. Among the more complex measures, Short-Interval Intracortical Inhibition, Silent Period [SP]), and Central Motor Conduction Time were rarely used, whereas Recruitment Curve, Long-Interval Intracortical Inhibition, and Intracortical Facilitation (ICF) were not assessed in any of the studies. Functional Magnetic Resonance Imaging (fMRI) was used in 11.4% (*N* = 15) of articles, while Electroencephalography (EEG) was only used in 5.3% (*N* = 7) of studies. Magnetoencephalography (MEG) and functional Near-Infrared Spectroscopy were used in 3% or fewer (*N* ≤ 3) of the included articles, while Positron Emission Tomography was not used at all. Among fMRI, EEG, and MEG studies, resting state was collected more often than task data. If task data were collected, the tasks differed across studies including peripheral nerve stimulation, wrist extension, finger tapping, and active or passive movement of the index finger.

### Which assessments are conducted together

Next, we asked which assessments tools were commonly performed together (Figure 3(e) for clinical trials SI Figure 2(e)). FMA-UE was often performed with a clinical global assessment tool, most commonly the NIHSS, but also the mRS and BI. Structural imaging was often combined with both the FMA-UE and the NIHSS, and MRI T1-weighted, MRI T2-weighted, and CT were often conducted in the same study.

### Follow-up assessment

**Figure 4.**
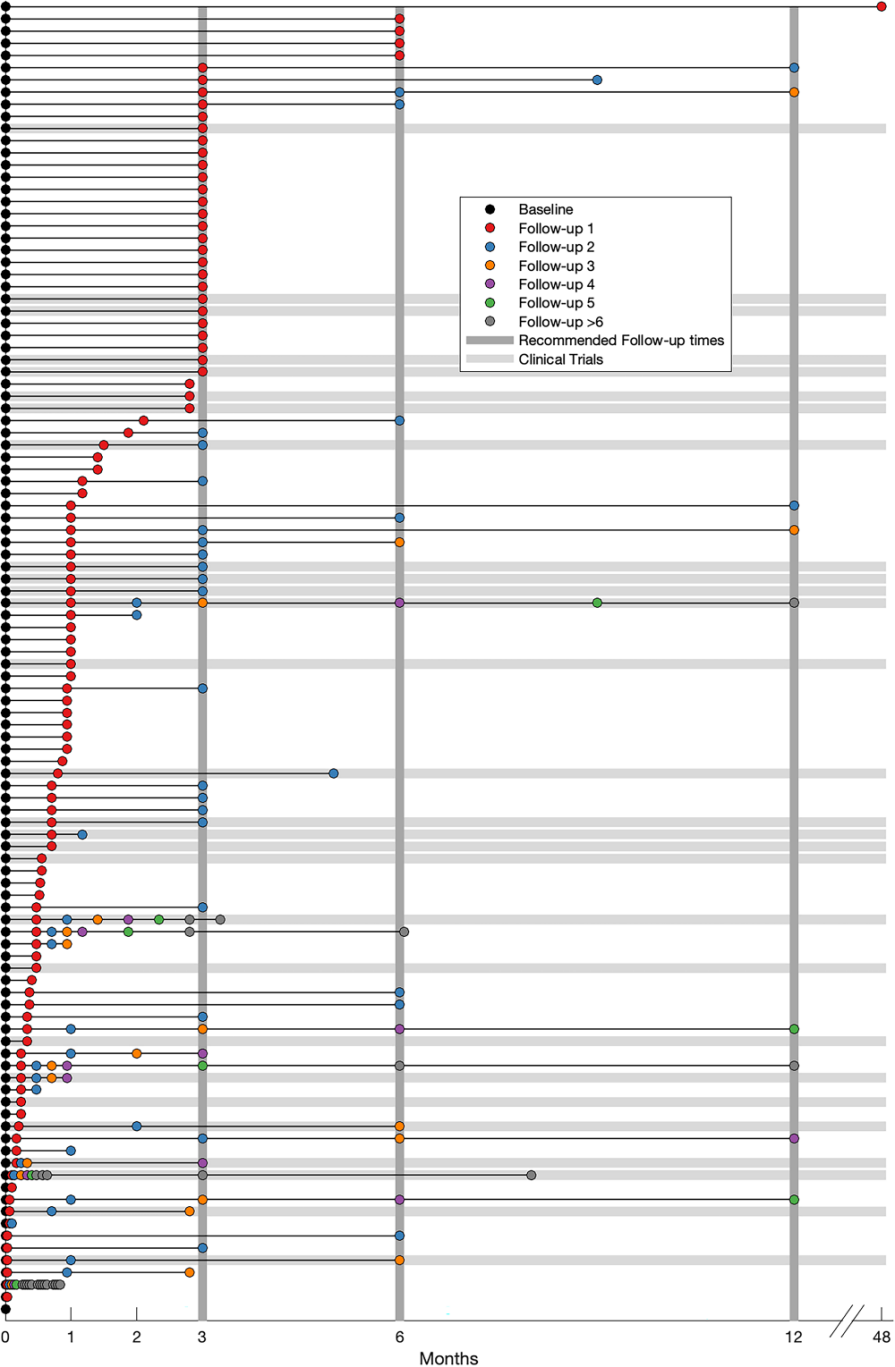
Timing of follow-up assessments. Each row represents one study in the scoping review, which are sorted based on the timing of the first follow-up. The baseline assessment is highlighted in black. Follow-up assessments 1–5 are represented by coloured circles, follow-up assessments larger than 5 are represented as grey circles. Grey horizontal lines highlight clinical trials. Grey vertical lines indicate the recommended time points for follow-up assessments.

81.8% (*N* = 108) of all studies (96.6% [*N* = 28] of clinical trials) conducted at least one follow-up assessment. Figure 4 depicts the distribution of clinical follow-up assessments across studies, measured at different intervals post-baseline. We found substantial variability regarding the timing of the follow-up assessments (range = 1 day to 4 years). Among the subset of clinical trials, only 46.4% (*N* = 13) conducted a follow-up at 3 months, which is strongly recommended.^15^ Following the 3-month mark, the frequency of follow-up assessments declines markedly, with only 9.8% (*N* = 13) of studies conducting follow-ups beyond 6 months.

## Discussion

This scoping review evaluated how current acute stroke research aligns with SRRR recommendations, specifically in assessing upper limb sensorimotor capacity. We found that global clinical measures were used fairly consistently, especially the NIHSS employed in over half of studies. The NIHSS remains a core tool for baseline severity^21–23^; however, its use as both a severity and outcome measure reflects misinterpretation of purpose.^15^ Other tools, the mRS and BI, were also frequent, assessing global disability or functional independence.^24,25^ This pattern mirrors findings from earlier trials (2001–2006), where the same three measures dominated.^26,27^ Their continued use reflects entrenched roles in evaluating stroke severity, independence, and disability.

The SRRR endorses a core set of validated clinical outcome measures to assess upper limb sensorimotor capacity.^15,16^ We found the FMA-UE was the most common motor outcome, its strong validation, and sensitivity to impairment-level change supporting widespread use.^15,^^28–30^ In contrast, relatively few studies employed the ARAT,^31,32^ which assesses activity limitations within the International Classification of Functioning, Disability and Health (ICF) framework.^33^ This imbalance between impairment and activity assessments suggests a focus on body function over daily-life performance. Across studies, 35 different upper limb measures were identified, revealing substantial heterogeneity. While this diversity reflects attempts to capture multiple dimensions of sensorimotor capacity,^34^ many studies used tools with limited validation despite available robust measures.^15^ Such inconsistency hinders data synthesis, limits comparability, and constrains meta-analysis.^35^ Greater consensus on validated tools covering both impairment and activity is needed to strengthen evidence quality. The SRRR also recommends kinematic assessments to complement clinical scales, as they can help differentiate between behavioural restitution and compensatory strategies.^16^ Although no acute-phase framework is specified, these methods are particularly valuable, as clinical scales may miss subtle motor changes. Kinematic assessments can detect fine movement-quality differences, offering insight into early recovery.^36–38^ Despite this promise, their use in acute stroke research remains limited, marking an opportunity for advancement.

The SRRR highlights biomarkers’ role in elucidating recovery mechanisms, recognising structural and functional measures for predicting outcomes, guiding interventions, and stratifying patients.^14^ We found structural neuroimaging was common, yet often used only for diagnostic confirmation rather than for outcome tracking or biomarker identification.^14^ Notably, advances now allow extraction of clinically relevant information even from standard clinical scans, expanding their potential.^39^ Greater emphasis on leveraging structural data for recovery stratification and monitoring brain changes could strengthen stroke rehabilitation research. Functional imaging was even less common, limited by cost, logistics, and variability in data acquisition and interpretation.^7,40^ However, this limited use restricts the depth of understanding regarding neural reorganisation and plasticity mechanisms following stroke. In line with the SRRR recommendations, broader adoption of functional imaging is essential for developing robust, reproducible biomarkers to improve prognosis and guide personalised rehabilitation.^14^

Our data suggest that only a limited number of studies incorporated multiple modalities. The lack of integrated assessments undermines the ability to understand the interaction between brain structure, brain function, and sensorimotor capacity.^41^ Future research should focus on multimodal designs that simultaneously evaluate clinical, structural, and functional metrics to develop a more holistic understanding of post-stroke recovery.^42,43^

While most studies included follow-up assessments, the timing varied widely. Although most conducted follow-up within 3 months, less than half of clinical trials included the SRRR mandated 3-month follow-up, which is considered essential for capturing meaningful recovery milestones.^15^ Few studies included 6- or 12-month assessments, despite the importance for tracking long-term outcomes.^15,44,45^ This variability may stem from logistical challenges, funding limitations, and participant retention concerns, but it introduces considerable noise into longitudinal comparisons.^46,47^

The SRRR recommends systematic reporting of baseline characteristics – age, sex, time since stroke onset, stroke severity, and imaging confirmations – to enable precise patient profiling.^15^ However, demographic details were often underreported. Fewer than one-third of studies included participants under the age of 35, and males were markedly overrepresented. This is concerning, as although stroke incidence is higher in men, women often experience greater severity and different recovery trajectories.^48–50^ The lack of gender balance aligns with prior research and raises concerns about the generalisability and potential bias of current findings.^51–53^ The SRRR emphasises biologically meaningful post-stroke time windows, e.g. acute (1–7 days).^5^ Yet, despite these definitions, interpretations remain inconsistent across studies and regions (e.g., ^54^) underscoring the need for global harmonisation of terminology and timing to improve assessment consistency and support robust data synthesis.

We focused on the SRRR guidelines as the primary benchmark for evaluating outcome measures due to their specific relevance to stroke recovery and rehabilitation, particularly in standardising assessment and research methodology. While other frameworks – such as those from NIH StrokeNet and the European Stroke Organisation – also inform study design and provide broader insights, the SRRR offer a more targeted complement closely aligned with our review goals. However, incorporating these frameworks may further enhance comprehensiveness. The review timeframe begins with the first SRRR publication; however, due to delays in guideline adoption, studies published soon after may not fully reflect recommendations, possibly contributing to lower alignment in early years. Further, while some measures are clearly described and easy to identify, others may appear under varying labels or as subtests within boarder tools, leading to potential omissions. Additionally, the acute phase presents clinical and practical limitations, making some assessments – especially those requiring active participation or complex movement – less feasible.^7^ In contrast, bedside assessments with minimal physical demands are often more practical and therefore more commonly used in acute stroke research.

## Supplemental Material

sj-docx-1-cre-10.1177_02692155251398368 - Supplemental material for Standardisation in acute stroke research: A scoping review of upper limb assessments against Stroke Recovery and Rehabilitation Roundtable (SRRR) benchmarks Supplemental material, sj-docx-1-cre-10.1177_02692155251398368 for Standardisation in acute stroke research: A scoping review of upper limb assessments against Stroke Recovery and Rehabilitation Roundtable (SRRR) benchmarks by Milica Doric, Lisa Tedesco Triccas, Mingyao Xiong, Faye Tabone, Adrian L Knorz, Nicole Downar, Nick S Ward and Catharina Zich in Clinical Rehabilitation
