# Supplementary material for: Standardisation in acute stroke research: A scoping review of upper limb assessments against Stroke Recovery and Rehabilitation Roundtable (SRRR) benchmarks
Source: Clin Rehabil. 2025 Nov 26;40(4):509–22. doi: 10.1177/02692155251398368 (PMC13013641; doi:10.1177/02692155251398368)
Supplement: sj-docx-1-cre-10.1177_02692155251398368 - Supplemental material for Standardisation in acute stroke research: A scoping review of upper limb assessments against Stroke Recovery and Rehabilitation Roundtable (SRRR) benchmarks [file sj-docx-1-cre-10.1177_02692155251398368.docx]

**Supplementary Material**

**Standardisation in Acute Stroke Research: A Scoping Review of Upper Limb Assessments Against Stroke Recovery Rehabilitation Roundtable Benchmarks**

Milica Doric^1^, Lisa Tedesco Triccas^1,2^, Mingyao Xiong^1^, Faye Tabone^3^, Adrian L Knorz^3^, Nicole Downar^3^, Nick S Ward^1^, Catharina Zich^1,3,4^

**Supplementary Methods**

**Supplementary Table 1**. *Search strategies limited to publications from 01.08.2017 - 30.09.2025*

| **Database** | **Platform** | **Search string** |
| --- | --- | --- |
| **Embase** | Ovid | *(“arm”:ti,ab,kw OR “upper limb”:ti,ab,kw OR “upper extremity”:ti,ab,kw OR “hand”:ti,ab,kw OR “wrist”:ti,ab,kw) AND (“movement”:ti,ab,kw OR “motor”:ti,ab,kw) AND (“acute”:ti,ab,kw OR “early”:ti,ab,kw) AND “stroke”:ti,ab,kw* |
| **MEDLINE** | Ovid | *(“arm”:ti,ab,kw OR “upper limb”:ti,ab,kw OR “upper extremity”:ti,ab,kw OR “hand”:ti,ab,kw OR “wrist”:ti,ab,kw) AND (“movement”:ti,ab,kw OR “motor”:ti,ab,kw) AND (“acute”:ti,ab,kw OR “early”:ti,ab,kw) AND “stroke”:ti,ab,kw* |
| **PubMed** | PubMed | *((("arm"[Title/Abstract] OR "upper limb"[Title/Abstract] OR "upper extremity"[Title/Abstract] OR "hand"[Title/Abstract] OR "wrist"[Title/Abstract]) AND "acute"[Title/Abstract]*  *AND "stroke"[Title/Abstract] AND "motor"[Title/Abstract])*  *NOT "sub-acute"[Title/Abstract]) NOT "subacute"[Title/Abstract])* |
| **CINAHL** | EBSCO | ("arm":TI,AB OR "upper limb":TI,AB OR "upper extremity":TI,AB OR "hand":TI,AB OR "wrist":TI,AB) AND ("motor":TI,AB OR "movement":TI,AB) AND ("acute":TI,AB AND "stroke":TI,AB NOT "subacute":TI,AB) |
| **PsycINFO** | Ovid | *(“arm”:ti,ab OR “upper limb”:ti,ab OR “upper extremity”:ti,ab OR “hand”:ti,ab OR “wrist”:ti,ab) AND (“movement”:ti,ab OR “motor”:ti,ab) AND (“acute”:ti,ab OR “early”:ti,ab) AND*  *“stroke”:ti,ab* |
| **Google Scholar** | Google Scholar | *allintitle: acute stroke motor arm OR limb OR extremity OR hand 0R wrist–subacute* |
| **Web of**  **Science** | Web of  Science | *((((((TI=(motor)) OR TI=(movement) AND TI=(arm) OR TI**=(upper limb) OR TI=(upper extremity) OR TI=(hand) OR TI=(wrist)* *AND TI=(stroke) AND TI=(acute)* |

**Comprehensive list of data extraction elements**

- **Paper Specifics**
  - Author
  - Year
  - Title
  - Journal
  - URL (DOI or stable link)
- **Study Type**
  - Interventional
    - Yes/No
    - Clinical trial (1/0)
    - Clinical trial phase
    - Clinical trial link
  - Observational
    - Longitudinal
    - Cross-sectional
    - Case report(s)
    - Other (specify)
- **Demographics**
  - Number of sites
  - Recruitment period
  - Sample size (N)
  - Male (n)
  - Female (n)
  - Age (mean, range)
  - Time post-stroke at inclusion
  - City
  - Country
  - Notes (e.g., median age, key cohort characteristics)
- **Clinical Assessments (1 = included, 0 = not included)**
  - **Upper Limb Motor**
    - Fugl-Meyer Assessment Upper Extremity (FMA-UE)
    - Action Research Arm Test (ARAT)
    - Box and Block Test (BBT)
    - Nine Hole Peg Test (NHPT)
    - Wolf Motor Function Test (WMFT)
    - Grip Strength
    - Medical Research Council (MRC) Scale
    - Lovett Scale
    - Ashworth Scale
    - Electromyography (EMG)
    - Chedoke Arm and Hand Activity Inventory (CAHAI)
    - Kinematic assessments
  - **Upper Limb Sensation**
    - PinPrick
    - Fugl-Meyer Sensory
    - Touch
  - **Global**
    - National Institutes of Health Stroke Scale (NIHSS)
    - Modified Rankin Scale (mRS)
    - Functional Independence Measure (FIM)
    - 36-item Short-Form Health Survey (SF-36)
    - EQ-5D
    - Barthel Index (BI)
    - Stroke Impact Scale (SIS)
- Notes (e.g. other tests, used for inclusion/exclusion criteria)
- **Clinical Assessments Follow-Up**
  - Yes/No
  - When
  - What
- **Brain Structure (1/0)**
  - Computed Tomography (CT)
  - MRI T1
  - MRI T2
  - MRI FLAIR
  - MRI DTI
  - MRI DWI
  - MRI Proton
  - MRI GRASE
  - Other (specify)
  - Notes
- **Brain Structure Follow-up**
  - Yes/No
  - When
  - What
- **Brain Function (1/0)**
  - Electroencephalography (EEG)
    - Rest
    - Task
    - Task category
  - Magnetoencephalography (MEG)
    - Rest
    - Task
    - Task category
  - Functional MRI (fMRI)
    - Rest
    - Task
    - Task category
  - Functional Near-Infrared Spectroscopy (fNIRS)
    - Rest
    - Task
    - Task category
  - Positron Emission Tomography (PET)
    - Rest
    - Task
    - Task category
  - Transcranial Magnetic Stimulation (TMS)
    - MEP
    - Motor Threshold
    - Recruitment curve
    - Short-Interval Intracortical Inhibition (SICI)
    - Silent Period (SP)
    - Long-Interval Intracortical Inhibition (LICI)
    - Intracortical Facilitation (ICF)
  - Other (specify)
  - Notes
- **Brain Function Follow-Up**
  - Yes/No
  - When
  - What
- **Other Biological Measures (1/0)**
  - Blood biomarkers
  - Genetic testing
  - Other (specify)

**Included Publications**

1. Abela, E., Missimer, J. H., Pastore-Wapp, M., Krammer, W., Wiest, R., & Weder, B. J. (2019). Early prediction of long-term tactile object recognition performance after sensorimotor stroke. Cortex; a journal devoted to the study of the nervous system and behavior, 115, 264-279. <https://doi.org/https://dx.doi.org/10.1016/j.cortex.2019.01.018>
2. Ahmad, S., Singh, V. K., Chaurasia, R. N., Mishra, V. N., Pathak, A., Kumar, A., Joshi, D., & Singh, G. (2025). Efficacy and Safety of Early Neuromuscular Electrical Stimulation on Shoulder and Arm Paresis in Patients of Acute Ischaemic Stroke-A Quasi-experimental Study. Annals of neurosciences, 09727531251365376. https://doi.org/https://dx.doi.org/10.1177/09727531251365376
3. Aikio, R., Laaksonen, K., Sairanen, V., Parkkonen, E., Abou Elseoud, A., Kujala, J., & Forss, N. (2021). CMC is more than a measure of corticospinal tract integrity in acute stroke patients. Neuroimage Clin, 32, 102818. <https://doi.org/10.1016/j.nicl.2021.102818>
4. Anandan, A. D., Selvaraj, S. K., Regan, R., Subramanian, S. S., Neelam, S., & Gaowgeh, R. A. M. (2021). Efficacy of Action Observation for Upper Limb Motor Deficit in Acute Stroke Participants. JOURNAL OF PHARMACEUTICAL RESEARCH INTERNATIONAL, 33(56A), 227-233. <https://doi.org/10.9734/JPRI/2021/v33i56A33905>
5. Andringa, A., Meskers, C., van de Port, I., van Wegen, E., & Kwakkel, G. (2020). Time Course of Wrist Hyper-Resistance in Relation to Upper Limb Motor Recovery Early Post Stroke. NEUROREHABILITATION AND NEURAL REPAIR, 34(8), 690-701. <https://doi.org/https://dx.doi.org/10.1177/1545968320932135>
6. Baguma, M., Yeganeh Doost, M., Riga, A., Laloux, P., Bihin, B., & Vandermeeren, Y. (2020). Preserved motor skill learning in acute stroke patients. Acta Neurol Belg, 120(2), 365-374. https://doi.org/10.1007/s13760-020-01304-7
7. Barth, J., Geed, S., Mitchell, A., Lum, P. S., Edwards, D. F., & Dromerick, A. W. (2020). Characterizing upper extremity motor behavior in the first week after stroke. PLoS One, 15(8), e0221668. <https://doi.org/https://dx.doi.org/10.1371/journal.pone.0221668>
8. Biykuzieva, A. A., Ahmedova, D. M., Usmankhodjaeva, A. A., Visogortseva, O. N., Svyatskaya, E. F., & Isabaeva, D. H. (2020). The role of robotic mechanotherapy in the recovery of mobility in patients after an acute cerebrovascular accident. Indian Journal of Forensic Medicine and Toxicology, 14(4), 7603 <https://doi.org/https://dx.doi.org/10.37506/ijfmt.v14i4.12851>
9. Boccuni, L., Meyer, S., D'Cruz, N., Kessner, S. S., Marinelli, L., Trompetto, C., Peeters, A., Van Pesch, V., Duprez, T., Sunaert, S., Feys, H., Thijs, V., Nieuwboer, A., & Verheyden, G. (2019). Premotor dorsal white matter integrity for the prediction of upper limb motor impairment after stroke. Scientific reports, 9(1), 19712. https://doi.org/https://dx.doi.org/10.1038/s41598-019-56334-w
10. Boccuni, L., Meyer, S., Kessner, S. S., De Bruyn, N., Essers, B., Cheng, B., Thomalla, G., Peeters, A., Sunaert, S., Duprez, T., Marinelli, L., Trompetto, C., Thijs, V., & Verheyden, G. (2018). Is There Full or Proportional Somatosensory Recovery in the Upper Limb After Stroke? Investigating Behavioral Outcome and Neural Correlates. NEUROREHABILITATION AND NEURAL REPAIR, 32(8), 691-700. https://doi.org/https://dx.doi.org/10.1177/1545968318787060
11. Bolognini, N., Russo, C., Souza Carneiro, M. I., Nicotra, A., Olgiati, E., Spandri, V., Agostoni, E., Salmaggi, A., & Vallar, G. (2020). Bi-hemispheric transcranial direct current stimulation for upper-limb hemiparesis in acute stroke: a randomized, double-blind, sham-controlled trial. Eur J Neurol, 27(12), 2473-2482. <https://doi.org/10.1111/ene.14451>
12. Bonkhoff, A. K., Espinoza, F. A., Gazula, H., Vergara, V. M., Hensel, L., Michely, J., Paul, T., Rehme, A. K., Volz, L. J., Fink, G. R., Calhoun, V. D., & Grefkes, C. (2020). Acute ischaemic stroke alters the brain's preference for distinct dynamic connectivity states. Brain, 143(5), 1525-1540. <https://doi.org/10.1093/brain/awaa101>
13. Bonkhoff, A. K., Rehme, A. K., Hensel, L., Tscherpel, C., Volz, L. J., Espinoza, F. A., Gazula, H., Vergara, V. M., Fink, G. R., Calhoun, V. D., Rost, N. S., & Grefkes, C. (2021). Dynamic connectivity predicts acute motor impairment and recovery post-stroke. Brain Commun, 3(4), fcab227. https://doi.org/10.1093/braincomms/fcab227
14. Bornheim, S., Croisier, J.-L., Maquet, P., & Kaux, J.-F. (2020). Transcranial direct current stimulation associated with physical-therapy in acute stroke patients - a randomized, triple blind, sham-controlled study [Neurological Disorders & Brain Damage 3297]. Brain Stimulation, 13(2), 329-336. <https://doi.org/https://dx.doi.org/10.1016/j.brs.2019.10.019>
15. Braga, M. A. F., Faria-Fortini, I., Dutra, T., Silva, E. A. M., Sant'Anna, R. V., & Faria, C. (2023). Functional independence measured in the acute phase of stroke predicts both generic and specific health-related quality of life: a 3-month prospective study in a middle-income country. Disabil Rehabil, 45(25), 4245-4251. <https://doi.org/10.1080/09638288.2022.2147590>
16. Branco, J. P., Oliveira, S., Sargento-Freitas, J., Galego, O., Cordeiro, G., Cunha, L., Gonçalves, A. F., & Pinheiro, J. (2019). Neuroimaging, serum biomarkers, and patient characteristics as predictors of upper limb functioning 12 weeks after acute stroke: an observational, prospective study. TOPICS IN STROKE REHABILITATION, 25(8), 584-590. <https://doi.org/10.1080/10749357.2018.1517491>
17. Bustren, E.-L., Sunnerhagen, K. S., & Alt Murphy, M. (2017). Movement Kinematics of the Ipsilesional Upper Extremity in Persons With Moderate or Mild Stroke. Neurorehabilitation and Neural Repair, 31(4), 376-386. <https://doi.org/https://dx.doi.org/10.1177/1545968316688798>
18. Cavanagh, S. K., Gochyyev, P., Nayeem, R., Dusang, A. N., Hamilton, T., DiCarlo, J. A., Kautz, S. A., Sternad, D., Walsh, C., Hochberg, L., & Lin, D. J. (2025). Trial-By-Trial Variation In Upper Extremity Movement Smoothness After Acute Stroke Relates To Clinical Assessments And Corticospinal Tract Injury. Neurorehabil Neural Repair, 39(8), 639-652. <https://doi.org/10.1177/15459683251340916>
19. Chatterjee, K., Stockley, R. C., Lane, S., Watkins, C., Cottrell, K., Ankers, B., Davies, S., Morris, M. F., Fallon, N., & Nurmikko, T. (2019). PULSE-I - Is rePetitive Upper Limb SEnsory stimulation early after stroke feasible and acceptable? A stratified single-blinded randomised controlled feasibility study. Trials, 20(1), 388. https://doi.org/10.1186/s13063-019-3428-y
20. Choi, W. (2022). The Effect of Task-Oriented Training on Upper-Limb Function, Visual Perception, and Activities of Daily Living in Acute Stroke Patients: A Pilot Study. Int J Environ Res Public Health, 19(6). https://doi.org/10.3390/ijerph19063186
21. Chunyong, L., Yingkai, L., Fuda, L., Jiang, C., & Liu, Y. (2023). Longitudinal changes of motor cortex function during motor recovery after stroke. Top Stroke Rehabil, 30(4), 342-354. https://doi.org/10.1080/10749357.2022.2051829
22. Coskunsu, D. K., Akcay, S., Ogul, O. E., Akyol, D. K., Ozturk, N., Zileli, F., Tuzun, B. B., & Krespi, Y. (2022). Effects of robotic rehabilitation on recovery of hand functions in acute stroke: A preliminary randomized controlled study. Acta Neurol Scand, 146(5), 499-511. <https://doi.org/10.1111/ane.13672>
23. Daghsen, L., Checkouri, T., Wittwer, A., Valabregue, R., Galanaud, D., Lejeune, F.-X., Doulazmi, M., Lamy, J.-C., Pouget, P., Roze, E., & Rosso, C. (2024). The relationship between corticospinal excitability and structural integrity in stroke patients. Journal of neurology, neurosurgery, and psychiatry, 96(1), 85-94. https://doi.org/https://dx.doi.org/10.1136/jnnp-2023-331996
24. Dalton, E. J., Jamwal, R., Augoustakis, L., Hill, E., Johns, H., Thijs, V., & Hayward, K. S. (2024). Prevalence of Arm Weakness, Pre-Stroke Outcomes and Other Post-Stroke Impairments Using Routinely Collected Clinical Data on an Acute Stroke Unit. Neurorehabil Neural Repair, 38(2), 148-160. https://doi.org/10.1177/15459683241229676
25. De Bruyn, N., Meyer, S., Kessner, S. S., Essers, B., Cheng, B., Thomalla, G., Peeters, A., Sunaert, S., Duprez, T., Thijs, V., Feys, H., Alaerts, K., & Verheyden, G. (2018). Functional network connectivity is altered in patients with upper limb somatosensory impairments in the acute phase post stroke: A cross-sectional study. PLoS One, 13(10), e0205693. https://doi.org/10.1371/journal.pone.0205693
26. Dehem, S., Gilliaux, M., Stoquart, G., Detrembleur, C., Jacquemin, G., Palumbo, S., Frederick, A., & Lejeune, T. (2019). Effectiveness of upper-limb robotic-assisted therapy in the early rehabilitation phase after stroke: A single-blind, randomised, controlled trial. Ann Phys Rehabil Med, 62(5), 313-320. https://doi.org/10.1016/j.rehab.2019.04.002
27. Delavaran, H., Aked, J., Sjunnesson, H., Lindvall, O., Norrving, B., Kokaia, Z., & Lindgren, A. (2017). Spontaneous Recovery of Upper Extremity Motor Impairment After Ischemic Stroke: Implications for Stem Cell-Based Therapeutic Approaches. Translational stroke research, 8(4), 351-361. https://doi.org/https://dx.doi.org/10.1007/s12975-017-0523-9
28. Du, J., Hu, J., Hu, J., Xu, Q., Zhang, Q., Liu, L., Ma, M., Xu, G., Zhang, Y., Liu, X., Lu, G., Zhang, Z., & Yang, F. (2018). Aberrances of Cortex Excitability and Connectivity Underlying Motor Deficit in Acute Stroke. Neural Plast, 2018, 1318093. <https://doi.org/10.1155/2018/1318093>
29. Edwardson, M. A., Nayak, A., Irfanoglu, M. O., Luby, M. L., Latour, L. L., & Pierpaoli, C. (2025). Association Between Changes in White Matter Volume Detected With Diffusion Tensor-Based Morphometry and Motor Recovery After Stroke. Neurology, 104(7), e213408. https://doi.org/https://dx.doi.org/10.1212/WNL.0000000000213408
30. Ekstrand, E., Sunnerhagen, K. S., Persson, H. C., Lundgren-Nilsson, A., & Alt Murphy, M. (2020). Longitudinal changes of self-perceived manual ability the first year after stroke: a cohort study. BMC neurology, 20(1), 181. <https://doi.org/https://dx.doi.org/10.1186/s12883-020-01754-9>
31. El Nahas, N., Roushdy, T. M., Shokri, H. M., Moustafa, R. R., Elsayed, A. M., Amin, R. M., Ashour, A. A., Abd Eldayem, E. H., Elhawary, G. A., & Elbokl, A. M. (2022). Lateralized readiness potentials can identify hemisphere of recovery in stroke patients. Restor Neurol Neurosci, 40(2), 63-71. <https://doi.org/10.3233/rnn-211222>
32. Engelter, S. T., Kaufmann, J. E., Zietz, A., Luft, A. R., Polymeris, A., Altersberger, V. L., Wiesner, K., Wiegert, M., Held, J. P. O., Rottenberger, Y., Schwarz, A., Medlin, F., Accolla, E. A., Foucras, S., Kägi, G., De Marchis, G. M., Politz, S., Greulich, M., Tarnutzer, A. A., Sturzenegger, R., Katan, M., Fischer, U., Nedeltchev, K., Schär, J., Van Den Keybus Deglon, K., Rapin, P. A., Salerno, A., Seiffge, D. J., Auer, E., Lippert, J., Bonati, L. H., Schuster-Amft, C., Gäumann, S., Chabwine, J. N., Humm, A., Möller, J. C., Schweinfurther, R., Bujan, B., Jedrysiak, P., Sandor, P. S., Gonzenbach, R., Mylius, V., Lutz, D., Lienert, C., Peters, N., Michel, P., Müri, R. M., Schädelin, S., Hemkens, L. G., Ford, G. A., Lyrer, P. A., Gensicke, H., & Traenka, C. (2025). Levodopa Added to Stroke Rehabilitation: The ESTREL Randomized Clinical Trial. Jama. https://doi.org/10.1001/jama.2025.15185
33. Esteki-Ghashghaei, F., Saadatnia, M., Khorvash, F., & Shahnazi, H. (2020). The Effect of Home Base Physical Activity Program based on the BASNEF Model on Motor Recovery in Patients with Stroke. Home Health Care Services Quarterly, 39(3), 154-167. https://doi.org/10.1080/01621424.2020.1765938
34. Feldner, H. A., Papazian, C., Peters, K. M., Creutzfeldt, C. J., & Steele, K. M. (2021). Clinical Use of Surface Electromyography to Track Acute Upper Extremity Muscle Recovery after Stroke: A Descriptive Case Study of a Single Patient. APPLIED SYSTEM INNOVATION, 4(2), Article 32. https://doi.org/10.3390/asi4020032
35. Fernández-Solana, J., Alvarez-Pardo, S., Moreno-Villanueva, A., Santamaría-Peláez, M., González-Bernal, J. J., Vélez-Santamaría, R., & González-Santos, J. (2024). Efficacy of a Rehabilitation Program Using Mirror Therapy and Cognitive Therapeutic Exercise on Upper Limb Functionality in Patients with Acute Stroke. HEALTHCARE, 12(5), Article 569. https://doi.org/10.3390/healthcare12050569
36. Gang, L., Shuangquan, T., Chao, D., Kangqiang, P., Chuanmiao, X., Shihui, X., Jinsheng, Z., Liu, G., Tan, S., Dang, C., Peng, K., Xie, C., Xing, S., & Zeng, J. (2017). Motor Recovery Prediction With Clinical Assessment and Local Diffusion Homogeneity After Acute Subcortical Infarction. Stroke (00392499), 48(8), 2121-2128. https://doi.org/10.1161/STROKEAHA.117.017060
37. Garrido M, M., Alvarez E, E., Acevedo P, F., Moyano V, A., Castillo N, N., & Cavada Ch, G. (2023). Early transcranial direct current stimulation with modified constraint-induced movement therapy for motor and functional upper limb recovery in hospitalized patients with stroke: A randomized, multicentre, double-blind, clinical trial [Neurological Disorders & Brain Damage 3297]. Brain Stimulation, 16(1), 40-47. https://doi.org/https://dx.doi.org/10.1016/j.brs.2022.12.008
38. Geng, W., Zhang, J., Shang, S., Chen, H., Shi, M., Jiang, L., Yin, X., & Chen, Y. C. (2022). Reduced functional network connectivity is associated with upper limb dysfunction in acute ischemic brainstem stroke. Brain Imaging Behav, 16(2), 802-810. <https://doi.org/10.1007/s11682-021-00554-0>
39. Gerardin, E., Regnier, M., Dricot, L., Lambert, J., van Ravestyn, C., De Coene, B., Bihin, B., Lindberg, P., & Vandermeeren, Y. (2024). Dexterity in the Acute Phase of Stroke: Impairments and Neural Substrates. Neurorehabil Neural Repair, 38(3), 229-239. https://doi.org/10.1177/15459683241230029
40. Glaess-Leistner, S., Ri, S. J., Audebert, H. J., & Wissel, J. (2021). Early clinical predictors of post stroke spasticity. TOPICS IN STROKE REHABILITATION, 28(7), 508-518. https://doi.org/10.1080/10749357.2020.1843845
41. Glize, B., Bigourdan, A., Villain, M., Munsch, F., Tourdias, T., de Gabory, I., Dehail, P., Dousset, V., Guehl, D., Joseph, P.-A., Laganaro, M., & Sibon, I. (2019). Motor evoked potential of upper-limbs is predictive of aphasia recovery. Aphasiology, 33(1), 105-120. https://doi.org/10.1080/02687038.2018.1444137
42. Guan, Y.-Z., Li, J., Zhang, X.-W., Wu, S., Du, H., Cui, L.-Y., & Zhang, W.-H. (2017). Effectiveness of repetitive transcranial magnetic stimulation (rTMS) after acute stroke: A one-year longitudinal randomized trial [Neurological Disorders & Brain Damage 3297]. CNS Neuroscience & Therapeutics, 23(12), 940-946. https://doi.org/https://dx.doi.org/10.1111/cns.12762
43. Guo, X. X., Fan, B. Y., & Mao, Y. Y. (2018). Effectiveness of neuromuscular electrical stimulation for wrist rehabilitation after acute ischemic stroke. Medicine, 97(38), Article e12299. https://doi.org/10.1097/MD.0000000000012299
44. Hagberg, G., Ihle-Hansen, H., Abzhandadze, T., Reinholdsson, M., Hansen, H. I., & Sunnerhagen, K. S. (2023). Prognostic value of acute National Institutes of Health Stroke Scale Items on disability: a registry study of first-ever stroke in the western part of Sweden. BMJ Open, 13(12), e080007. <https://doi.org/10.1136/bmjopen-2023-080007>
45. Harquel, S., Cadic-Melchior, A., Morishita, T., Fleury, L., Ceroni, M., Menoud, P., Brugger, J., Beanato, E., Meyer, N. H., Evangelista, G. G., Egger, P., Van de Ville, D., Blanke, O., Micera, S., Leger, B., Adolphsen, J., Jagella, C., Muhl, A., Constantin, C., Alvarez, V., Vuadens, P., Turlan, J. L., Bonvin, C., Koch, P. J., Wessel, M. J., & Hummel, F. C. (2025). Brain Oscillatory Modes as a Proxy of Stroke Recovery. Neurorehabilitation and Neural Repair, 15459683251363241. <https://doi.org/https://dx.doi.org/10.1177/15459683251363241>
46. Harquel, S., Cadic-Melchior, A., Morishita, T., Fleury, L., Witon, A., Ceroni, M., Brugger, J., Meyer, N. H., Evangelista, G. G., Egger, P., Beanato, E., Menoud, P., Van De Ville, D., Micera, S., Blanke, O., Leger, B., Adolphsen, J., Jagella, C., Constantin, C., Alvarez, V., Vuadens, P., Turlan, J. L., Muhl, A., Bonvin, C., Koch, P. J., Wessel, M. J., & Hummel, F. C. (2024). Stroke Recovery-Related Changes in Cortical Reactivity Based on Modulation of Intracortical Inhibition. Stroke, 55(6), 1629 https://doi.org/https://dx.doi.org/10.1161/STROKEAHA.123.045174
47. Hodgson, K., Adluru, G., Richards, L. G., Majersik, J. J., Stoddard, G., Adluru, N., & Di Bella, E. (2019). Predicting motor outcomes in stroke patients using diffusion spectrum MRI microstructural measures. Frontiers in neurology, 10(FEB), 72. <https://doi.org/https://dx.doi.org/10.3389/fneur.2019.00072>
48. Hoonhorst, M. H. J., Nijland, R. H. M., Emmelot, C. H., Kollen, B. J., & Kwakkel, G. (2021). TMS-Induced Central Motor Conduction Time at the Non-Infarcted Hemisphere Is Associated with Spontaneous Motor Recovery of the Paretic Upper Limb after Severe Stroke. Brain sciences, 11(5). https://doi.org/https://dx.doi.org/10.3390/brainsci11050648
49. Hoonhorst, M. H. J., Nijland, R. H. M., van den Berg, P. J. S., Emmelot, C. H., Kollen, B. J., & Kwakkel, G. (2018). Does Transcranial Magnetic Stimulation Have an Added Value to Clinical Assessment in Predicting Upper-Limb Function Very Early After Severe Stroke? NEUROREHABILITATION AND NEURAL REPAIR, 32(8), 682-690. https://doi.org/https://dx.doi.org/10.1177/1545968318785044
50. Hosp, J. A., Dressing, A., Engesser, A., Glauche, V., Kümmerer, D., Vaidelyte, E. B., Musso, M., Rijntjes, M., & Weiller, C. (2023). The Role of Ascending Ventral-Tegmental Fibers for Recovery after Stroke. Ann Neurol, 93(5), 922-933. https://doi.org/10.1002/ana.26595
51. Hosseini, Z.-S., Peyrovi, H., & Gohari, M. (2019). The Effect of Early Passive Range of Motion Exercise on Motor Function of People with Stroke: a Randomized Controlled Trial. Journal of caring sciences, 8(1), 39-44. <https://doi.org/https://dx.doi.org/10.15171/jcs.2019.006>
52. Hu, J., Du, J., Xu, Q., Yang, F., Zeng, F., Dai, X., Liu, X., Lu, G., & Zhang, Z. (2017). Altered coupling between motion-related activation and resting-state brain activity in the ipsilesional sensorimotor cortex after cerebral stroke. Frontiers in Neurology, 8(JUL), 339. https://doi.org/https://dx.doi.org/10.3389/fneur.2017.00339
53. Jain, M., Harjpal, P., Kovela, R. K., & Vardhan, V. (2022). Positive Outcomes of Early Task-Specific Training and Action Observation Mirror Therapy Following Infarction of Hand Knob Area: A Case Report. Cureus, 14(10), e29819. <https://doi.org/10.7759/cureus.29819>
54. Jia, W., Zhou, Y., Mao, J., Feng, J., Han, Y., Xu, F., Wang, X., Liu, T., & Li, Z. (2025). Inhibition of Ipsilesional M1 β Oscillations by Contralesional M1 Following Acute Ischemic Stroke: A TMS-EEG Study. Stroke, 56(8), 2045-2056. https://doi.org/10.1161/strokeaha.124.050376
55. Jong-Hoon, M., Kyoung-Young, P., Hee-Jin, K., & Chang-Ho, N. (2018). The Effects of Task-Oriented Circuit Training Using Rehabilitation Tools on the Upper-Extremity Functions and Daily Activities of Patients with Acute Stroke: A Randomized Controlled Pilot Trial. Osong Public Health & Research Perspectives, 9(5), 225-230. https://doi.org/10.24171/j.phrp.2018.9.5.03
56. Jung, K. M., & Choi, J. D. (2019). The Effects of Active Shoulder Exercise with a Sling Suspension System on Shoulder Subluxation, Proprioception, and Upper Extremity Function in Patients with Acute Stroke. MEDICAL SCIENCE MONITOR, 25, 4849-4855. https://doi.org/10.12659/MSM.915277
57. Karaahmet, O. Z., Umay, E., Gurcay, E., Serçe, A., Gundogdu, I., & Cakci, A. (2018). The effect of premorbid features on post-stroke rehabilitation outcome. Iran J Neurol, 17(1), 38-46.
58. Ke, J., Zou, X., Huang, M., Huang, Q., Li, H., & Zhou, X. (2020). High-frequency rTMS with two different inter-train intervals improves upper limb motor function at the early stage of stroke. Journal of International Medical Research, 48(6). https://doi.org/https://dx.doi.org/10.1177/0300060520928737
59. Kessner, S. S., Schlemm, E., Cheng, B., Bingel, U., Fiehler, J., Gerloff, C., & Thomalla, G. (2019). Somatosensory Deficits After Ischemic Stroke. Stroke, 50(5), 1116-1123. <https://doi.org/10.1161/strokeaha.118.023750>
60. Kim, D., Ko, S.-H., Han, J., Kim, Y.-T., Kim, Y.-H., Chang, W. H., & Shin, Y.-I. (2024). Correlations in abnormal synergies between the upper and lower extremities across various phases of stroke. Journal of Neurophysiology, 132(1), 87-95. https://doi.org/https://dx.doi.org/10.1152/jn.00102.2024 (Update of: medRxiv. 2024 Feb 09:2024.02.07.24302477. doi: 10.1101/2024.02.07.24302477 PMID: 38370652 [https://pubmed.ncbi.nlm.nih.gov/38370652])
61. Kumar, P., Prasad, M., Das, A., Vibha, D., Garg, A., Goyal, V., & Srivastava, A. (2022). Utility of Transcranial Magnetic Stimulation and Diffusion Tensor maging for Prediction of Upper-Limb Motor Recovery n Acute Ischemic Stroke Patients. ANNALS OF INDIAN ACADEMY OF NEUROLOGY, 25(1), 54-59. https://doi.org/10.4103/aian.aian_254_21
62. Lee, S. H., Song, B. K., & Kim, H. N. (2019). The effect of bilateral upper limb training on recovery oupper limb function in patients with acute stroke. Medico-Legal Update, 19(1), 515-520. https://doi.org/https://dx.doi.org/10.5958/0974-1283.2019.00095.1
63. Li, Y., Wang, Q., Liu, X.-L., Hui, R., & Zhang, Y.-P. (2023). Effect of the physical rehabilitation program based on self-care ability in patients with acute ischemic stroke: a quasi-experimental study. Frontiers in neurology, 14, 1181651. https://doi.org/https://dx.doi.org/10.3389/fneur.2023.1181651
64. Lin, D. J., Cloutier, A. M., Erler, K. S., Cassidy, J. M., Snider, S. B., Ranford, J., Parlman, K., Giatsidis, F., Burke, J. F., & Schwamm, L. (2020). Corticospinal Tract Injury Estimated From Acute Stroke Imaging Predicts Upper Extremity Motor Recovery After Stroke. Stroke, 51(Suppl_1), A13-A13.
65. Lin, D. J., Erler, K. S., Snider, S. B., Bonkhoff, A. K., DiCarlo, J. A., Lam, N., Ranford, J., Parlman, K., Cohen, A., & Freeburn, J. (2021). Cognitive demands influence upper extremity motor performance during recovery from acute stroke. Neurology, 96(21), e2576-e2586.
66. Lin, D. J., Hardstone, R., DiCarlo, J. A., McKiernan, S., Snider, S. B., Jacobs, H., Erler, K. S., Rishe, K., Boyne, P., Goldsmith, J., Ranford, J., Finklestein, S. P., Schwamm, L. H., Hochberg, L. R., & Cramer, S. C. (2023). Distinguishing Distinct Neural Systems for Proximal vs Distal Upper Extremity Motor Control After Acute Stroke. Neurology, 101(4), e347-e357. https://doi.org/10.1212/wnl.0000000000207417
67. Lin, R., Hsu, M.-J., Lin, R.-T., Huang, M.-H., Koh, C.-L., Hsieh, C.-L., & Lin, J.-H. (2017). No Difference Between Noxious and Innocuous Thermal Stimulation on Motor Recovery of Upper Extremity in Patients With Acute Stroke: A Randomized Controlled Trial With 6-Month Follow-up. PM & R : the journal of injury, function, and rehabilitation, 9(12), 1191-1199. https://doi.org/https://dx.doi.org/10.1016/j.pmrj.2017.05.012
68. Liu, G., Wu, J., Dang, C., Tan, S., Peng, K., Guo, Y., Xing, S., Xie, C., Zeng, J., & Tang, X. (2022). Machine Learning for Predicting Motor Improvement After Acute Subcortical Infarction Using Baseline Whole Brain Volumes. Neurorehabil Neural Repair, 36(1), 38-48. https://doi.org/10.1177/15459683211054178
69. Lubart, A. A., Leibovitz, A. I., Peleg, L. D., Yarovoy, A. I., Gal, G. O., Mizrahi, E. H., & Lubart, E. N. (2017). Action Observation of Motor Skills Followed by Immediate Sleep Enhances the Motor Rehabilitation of Older Adults With Stroke. Journal of geriatric physical therapy (2001). <https://doi.org/https://dx.doi.org/10.1519/JPT.0000000000000136>
70. Lv, S., Ran, X., Xia, M., Zhang, Y., Pang, T., Zhou, X., Zhao, Z., Yu, Y., & Gao, Z. (2025). Classification of left and right-hand motor imagery in acute stroke patients using EEG microstate. J Neuroeng Rehabil, 22(1), 137. https://doi.org/10.1186/s12984-025-01668-y
71. Malmut, L., Lin, C., Srdanovic, N., Kocherginsky, M., Harvey, R. L., & Prabhakaran, S. (2020). Arm Subscore of Motricity Index to Predict Recovery of Upper Limb Dexterity in Patients With Acute Ischemic Stroke. AMERICAN JOURNAL OF PHYSICAL MEDICINE & REHABILITATION, 99(4), 300-304. https://doi.org/10.1097/PHM.0000000000001326
72. Meng, G., Meng, X., Tan, Y., Yu, J., Jin, A., Zhao, Y., & Liu, X. (2017). Short-term Efficacy of Hand-Arm Bimanual Intensive Training on Upper Arm Function in Acute Stroke Patients: A Randomized Controlled Trial. Front Neurol, 8, 726. https://doi.org/10.3389/fneur.2017.00726
73. Molle Da Costa, R. D., Luvizutto, G. J., Martins, L. G., Thomaz De Souza, J., Regina Da Silva, T., Alvarez Sartor, L. C., Winckler, F. C., Modolo, G. P., Molle, E., Dos Anjos, S. M., Bazan, S. G. Z., Cuadrado, L. M., & Bazan, R. (2019). Clinical factors associated with the development of nonuse learned after stroke: a prospective study. Top Stroke Rehabil, 26(7), 511-517. https://doi.org/10.1080/10749357.2019.1631605
74. Moon, H. S., Heffron, L., Mahzarnia, A., Obeng-Gyasi, B., Holbrook, M., Badea, C. T., Feng, W., & Badea, A. (2022). Automated multimodal segmentation of acute ischemic stroke lesions on clinical MR images. Magn Reson Imaging, 92, 45-57. https://doi.org/10.1016/j.mri.2022.06.001
75. Moon, J.-H., Park, K.-Y., Kim, H.-J., & Na, C.-H. (2018). The Effects of Task-Oriented Circuit Training Using Rehabilitation Tools on the Upper-Extremity Functions and Daily Activities of Patients with Acute Stroke: A Randomized Controlled Pilot Trial. Osong public health and research perspectives, 9(5), 225-230. https://doi.org/https://dx.doi.org/10.24171/j.phrp.2018.9.5.03
76. Mostajeran, M., Alizadeh, S., Rostami, H. R., Ghaffari, A., & Adibi, I. (2023). Feasibility and efficacy of an early sensory-motor rehabilitation program on hand function in patients with stroke: a pilot, single-subject experimental design. Neurol Sci. <https://doi.org/10.1007/s10072-023-07288-5>
77. Nakashima, A., Koizumi, T., Shimizu, T., Ryu, N., & Higashi, T. (2024). Efficacy of mental practice on paralyzed upper extremity function in the acute phase of stroke: a case study. Journal of physical therapy science, 36(6), 364-366. https://doi.org/https://dx.doi.org/10.1589/jpts.36.364
78. Nemati, P. R., Backhaus, W., Feldheim, J., Bonstrup, M., Cheng, B., Thomalla, G., Gerloff, C., & Schulz, R. (2022). Brain network topology early after stroke relates to recovery [Neurological Disorders & Brain Damage 3297]. Brain Communications, 4(2). https://doi.org/https://dx.doi.org/10.1093/braincomms/fcac049
79. Obayashi, S., & Takahashi, R. (2020). Repetitive peripheral magnetic stimulation improves severe upper limb paresis in early acute phase stroke survivors. NeuroRehabilitation, 46(4), 569-575. <https://doi.org/10.3233/nre-203085>
80. Obayashi, S., Takahashi, R., & Onuki, M. (2020). Upper limb recovery in early acute phase stroke survivors by coupled EMG-triggered and cyclic neuromuscular electrical stimulation. NeuroRehabilitation, 46(3), 417-422. <https://doi.org/10.3233/nre-203024>
81. Oey, N. E., Samuel, G. S., Lim, J. K. W., VanDongen, A. M., Ng, Y. S., & Zhou, J. (2019). Whole Brain White Matter Microstructure and Upper Limb Function: Longitudinal Changes in Fractional Anisotropy and Axial Diffusivity in Post-Stroke Patients. Journal of central nervous system disease, 11, 1179573519863428. <https://doi.org/https://dx.doi.org/10.1177/1179573519863428>
82. Olczak, A., & Truszczyńska-Baszak, A. (2022). Motor Coordination and Grip Strength of the Dominant and Non-Dominant Affected Upper Limb Depending on the Body Position-An Observational Study of Patients after Ischemic Stroke. Brain Sci, 12(2). <https://doi.org/10.3390/brainsci12020164>
83. Oveisgharan, S., Organji, H., & Ghorbani, A. (2018). Enhancement of Motor Recovery through Left Dorsolateral Prefrontal Cortex Stimulation after Acute Ischemic Stroke. J Stroke Cerebrovasc Dis, 27(1), 185-191. <https://doi.org/10.1016/j.jstrokecerebrovasdis.2017.08.026>
84. Papazian, C., Baicoianu, N., Peters, K., Feldner, H., & Steele, K. (2019). Electromyography Recordings Reveal Muscle Activity in Flaccid Arm During Initial Days After Stroke. Archives of Physical Medicine and Rehabilitation, 100(12), e178. https://doi.org/https://dx.doi.org/10.1016/j.apmr.2019.10.047
85. Park, Y. S., An, C. S., & Lim, C. G. (2021). Effects of a Rehabilitation Program Using a Wearable Device on the Upper Limb Function, Performance of Activities of Daily Living, and Rehabilitation Participation in Patients with Acute Stroke. INTERNATIONAL JOURNAL OF ENVIRONMENTAL RESEARCH AND PUBLIC HEALTH, 18(11), Article 5524. <https://doi.org/10.3390/ijerph18115524>
86. Parkkonen, E., Laaksonen, K., Piitulainen, H., Pekkola, J., Parkkonen, L., Tatlisumak, T., & Forss, N. (2017). Strength of ~20-Hz Rebound and Motor Recovery After Stroke. Neurorehabil Neural Repair, 31(5), 475-486. <https://doi.org/10.1177/1545968316688795>
87. Patel, J., Fluet, G., Qiu, Q., Yarossi, M., Merians, A., Tunik, E., & Adamovich, S. (2019). Intensive virtual reality and robotic based upper limb training compared to usual care, and associated cortical reorganization, in the acute and early sub-acute periods post-stroke: a feasibility study. JOURNAL OF NEUROENGINEERING AND REHABILITATION, 16(1), 92. <https://doi.org/https://dx.doi.org/10.1186/s12984-019-0563-3>
88. Patel, J., Qiu, Q., Yarossi, M., Merians, A., Massood, S., Tunik, E., Adamovich, S., & Fluet, G. (2017). Exploring the impact of visual and movement based priming on a motor intervention in the acute phase post-stroke in persons with severe hemiparesis of the upper extremity. Disabil Rehabil, 39(15), 1515-1523. <https://doi.org/10.1080/09638288.2016.1226419>
89. Paul, T., Wiemer, V. M., Gunther, J., Lehnberg, F. M., Grafton, S. T., Fink, G. R., & Volz, L. J. (2024). Reward enhances motor adaptation learning in acute stroke patients. medRxiv. https://doi.org/https://dx.doi.org/10.1101/2024.04.03.24305243
90. Pinheiro, D. R. D., Cabeleira, M. E. P., da Campo, L. A., Gattino, L. A. F., de Souza, K. S., Burg, L. D., Blauth, A., Corrêa, P. S., & Cechetti, F. (2021). Upper limbs cycle ergometer increases muscle strength, trunk control and independence of acute stroke subjects: A randomized clinical trial. NeuroRehabilitation, 48(4), 533-542. <https://doi.org/10.3233/NRE-210022>
91. Rabadi, M. H., & Aston, C. E. (2017). Effect of Transcranial Direct Current Stimulation on Severely Affected Arm-Hand Motor Function in Patients After an Acute Ischemic Stroke: A Pilot Randomized Control Trial. Am J Phys Med Rehabil, 96(10 Suppl 1), S178-s184. <https://doi.org/10.1097/phm.0000000000000823>
92. Riga, A., Gathy, E., Ghinet, M., De Laet, C., Bihin, B., Regnier, M., Leeuwerck, M., De Coene, B., Dricot, L., Herman, B., Edwards, M. G., & Vandermeeren, Y. (2022). Evidence of Motor Skill Learning in Acute Stroke Patients Without Lesions to the Thalamus and Internal Capsule. Stroke, 53(7), 2361-2368. <https://doi.org/10.1161/strokeaha.121.035494>
93. Rosso, C., Daghsen, L., Bouvier, J., Checkouri, T., Millot, S., Baronnet, F., Galanaud, D., Valabregue, R., Pouget, P., Lamy, J.-C., & Roze, E. (2025). Could the Early Disinhibition of the Unaffected Motor Cortex Predict Motor Recovery After Stroke? Stroke, 56(10), 2904-2913. https://doi.org/https://dx.doi.org/10.1161/STROKEAHA.125.051614
94. Saadatnia, M., Shahnazi, H., Khorvash, F., & Esteki-Ghashghaei, F. (2020). The Impact of Home-Based Exercise Rehabilitation on Functional Capacity in Patients With Acute Ischemic Stroke: A Randomized Controlled Trial. Home Health Care Management & Practice, 32(3), 141-147. <https://doi.org/10.1177/1084822319895982>
95. Saes, M., Mohamed Refai, M. I., van Kordelaar, J., Scheltinga, B. L., van Beijnum, B.-J. F., Bussmann, J. B. J., Buurke, J. H., Veltink, P. H., Meskers, C. G. M., van Wegen, E. E. H., & Kwakkel, G. (2021). Smoothness metric during reach-to-grasp after stroke: part 2. longitudinal association with motor impairment. JOURNAL OF NEUROENGINEERING AND REHABILITATION, 18(1), 144. <https://doi.org/https://dx.doi.org/10.1186/s12984-021-00937-w>
96. Saito, J., Koyama, T., & Domen, K. (2018). Long-Term Outcomes of FIM Motor Items Predicted From Acute Stage NIHSS of Patients With Middle Cerebral Artery Infarct. Ann Rehabil Med, 42(5), 670-681. <https://doi.org/10.5535/arm.2018.42.5.670>
97. Sakurada, T., Goto, A., Tetsuka, M., Nakajima, T., Morita, M., Yamamoto, S.-I., Hirai, M., & Kawai, K. (2019). Prefrontal activity predicts individual differences in optimal attentional strategy for preventing motor performance decline: a functional near-infrared spectroscopy study. Neurophotonics, 6(2), 025012. https://doi.org/https://dx.doi.org/10.1117/1.NPh.6.2.025012
98. Sakurada, T., Nakajima, T., Morita, M., Hirai, M., & Watanabe, E. (2017). Improved motor performance in patients with acute stroke using the optimal individual attentional strategy. Scientific reports, 7, 40592. https://doi.org/https://dx.doi.org/10.1038/srep40592
99. Saltao da Silva, M. A., Baune, N. A., Belagaje, S., & Borich, M. R. (2022). Clinical Imaging-Derived Metrics of Corticospinal Tract Structural Integrity Are Associated With Post-stroke Motor Outcomes: A Retrospective Study. Frontiers in neurology, 13, 804133. <https://doi.org/https://dx.doi.org/10.3389/fneur.2022.804133>
100. Samuel, G. S., Oey, N. E., Choo, M., Ju, H., Chan, W. Y., Kok, S., Ge, Y., Van Dongen, A. M., & Ng, Y. S. (2017). Combining levodopa and virtual reality-based therapy for rehabilitation of the upper limb after acute stroke: pilot study Part II. Singapore Med J, 58(10), 610-617. <https://doi.org/10.11622/smedj.2016111>
101. Schlemm, E., Schulz, R., Bönstrup, M., Krawinkel, L., Fiehler, J., Gerloff, C., Thomalla, G., & Cheng, B. (2020). Structural brain networks and functional motor outcome after stroke-a prospective cohort study. Brain Commun, 2(1), fcaa001. <https://doi.org/10.1093/braincomms/fcaa001>
102. Shibata, T., Urata, A., Kawahara, K., Furuya, K., Ishikuro, K., Hattori, N., & Kuroda, S. (2020). Therapeutic Effects of Diagonal-Transcranial Direct Current Stimulation on Functional Recovery in Acute Stroke: A Pilot Study. Journal of stroke and cerebrovascular diseases : the official journal of National Stroke Association, 29(10), 105107. <https://doi.org/https://dx.doi.org/10.1016/j.jstrokecerebrovasdis.2020.105107>
103. Snickars, J., Persson, H. C., & Sunnerhagen, K. S. (2017). Early clinical predictors of motor function in the upper extremity one month post-stroke. Journal of rehabilitation medicine, 49(3), 216-222. <https://doi.org/https://dx.doi.org/10.2340/16501977-2205>
104. Spampinato, M. V., Chan, C., Jensen, J. H., Helpern, J. A., Bonilha, L., Kautz, S. A., Nietert, P. J., & Feng, W. (2017). Diffusional Kurtosis Imaging and Motor Outcome in Acute Ischemic Stroke. AJNR Am J Neuroradiol, 38(7), 1328-1334. <https://doi.org/10.3174/ajnr.A5180>
105. Sung Ho, J., Jun, L., You Sung, S., Jang, S. H., Lee, J., & Seo, Y. S. (2020). Motor recovery by the aberrant pyramidal pathway in a patient with cerebral infarct. Medicine, 99(22), 1-4. <https://doi.org/10.1097/MD.0000000000020282>
106. Topcuoglu, M. A., Rocha, E. A., Siddiqui, A. K., Mills, B. B., Silva, G. S., Schwamm, L. H., Lamuraglia, G. M., & Singhal, A. B. (2018). Isolated Upper Limb Weakness From Ischemic Stroke: Mechanisms and Outcome. J Stroke Cerebrovasc Dis, 27(10), 2712-2719. https://doi.org/10.1016/j.jstrokecerebrovasdis.2018.05.050
107. Tscherpel, C., Hensel, L., Lemberg, K., Vollmer, M., Volz, L. J., Fink, G. R., & Grefkes, C. (2020). The differential roles of contralesional frontoparietal areas in cortical reorganization after stroke. Brain stimulation, 13(3), 614-624. https://doi.org/https://dx.doi.org/10.1016/j.brs.2020.01.016
108. Uchida, J., Yamada, M., Nagayama, H., Tomori, K., Ikeda, K., & Yamauchi, K. (2025). Classifying Patient Characteristics and Determining a Predictor in Acute Stroke Patients: Application of Latent Class Analysis in Rehabilitation Practice. J Clin Med, 14(15). https://doi.org/10.3390/jcm14155466
109. Ueda, S., Aoki, H., Yasuda, Y., Nishiyama, A., Hayashi, Y., Honaga, K., Tanuma, A., Takakura, T., Kurosu, A., Hatori, K., Hayashi, A., & Fujiwara, T. (2022). The MMT of Elbow Flexion and the AFE Predict Impairment and Disability at 3 Weeks in Patients With Acute Stroke. Front Neurol, 13, 831800. <https://doi.org/10.3389/fneur.2022.831800>
110. Umeki, N., Murata, J., & Higashijima, M. (2019). Effects of Training for Finger Perception on Functional Recovery of Hemiplegic Upper Limbs in Acute Stroke Patients. Occup Ther Int, 2019, 6508261. <https://doi.org/10.1155/2019/6508261>
111. van Assche, M., Dirren, E., Bourgeois, A., Kleinschmidt, A., Richiardi, J., & Carrera, E. (2021). Periinfarct rewiring supports recovery after primary motor cortex stroke. Journal of cerebral blood flow and metabolism : official journal of the International Society of Cerebral Blood Flow and Metabolism, 41(9), 2174-2184. <https://doi.org/https://dx.doi.org/10.1177/0271678X211002968>
112. Vanbellingen, T., Van de Winckel, A., Pastore-Wapp, M., Ottiger, B., Veerbeek, J., Cazzoli, D., & Nyffeler, T. (2025). Poor upper limb performance despite the absence of notable upper limb motor impairment in adults with acute stroke - the influence of cognitive deficits. Neuropsychol Rehabil, 1-11. https://doi.org/10.1080/09602011.2025.2541096
113. Vimolratana, O., Aneksan, B., Klamruen, P., Hiengkaew, V., Siripornpanich, V., & Klomjai, W. (2023). Effects of anodal transcranial direct current stimulation on upper extremity functions and brain activity in acute stroke patients. NEUROREHABILITATION AND NEURAL REPAIR, 37(5), NP120. <https://doi.org/https://dx.doi.org/10.1177/15459683231159499> (12th World Congress for Neurorehabilitation, WCNR 2022. Vienna Austria.)
114. Vratsistas-Curto, A., Downie, A., McCluskey, A., & Sherrington, C. (2023). Trajectories of arm recovery early after stroke: an exploratory study using latent class growth analysis. Annals of medicine, 55(1), 253-265. <https://doi.org/https://dx.doi.org/10.1080/07853890.2022.2159062>
115. Wang, D., & Dai, J. (2022). Intelligent Algorithm-Based MRI Image Features for Evaluating the Effect of Nursing on Recovery of the Neurological Function of Patients with Acute Stroke. Contrast Media Mol Imaging, 2022, 3936655. <https://doi.org/10.1155/2022/3936655>
116. Wang, F., Zhang, S., Zhou, F., Zhao, M., & Zhao, H. (2022). Early physical rehabilitation therapy between 24 and 48 h following acute ischemic stroke onset: a randomized controlled trial. Disabil Rehabil, 44(15), 3967-3972. <https://doi.org/10.1080/09638288.2021.1897168>
117. Wang, J., Zhang, Y., Chen, Y., Li, M., Yang, H., Chen, J., Tang, Q., & Jin, J. (2021). Effectiveness of Rehabilitation Nursing versus Usual Therapist-Led Treatment in Patients with Acute Ischemic Stroke: A Randomized Non-Inferiority Trial. Clin Interv Aging, 16, 1173-1184. <https://doi.org/10.2147/cia.S306255>
118. Wang, L., Xu, X., Kai Lau, K., Li, L. S. W., Kwun Wong, Y., Yau, C., Mak, H. K. F., & Hui, E. S. (2021). Relation between rich-club organization versus brain functions and functional recovery after acute ischemic stroke. Brain Res, 1763, 147441. <https://doi.org/10.1016/j.brainres.2021.147441>
119. Watanabe, K., Kudo, Y., Sugawara, E., Nakamizo, T., Amari, K., Takahashi, K., Tanaka, O., Endo, M., Hayakawa, Y., & Johkura, K. (2018). Comparative study of ipsilesional and contralesional repetitive transcranial magnetic stimulations for acute infarction. J Neurol Sci, 384, 10-14. <https://doi.org/10.1016/j.jns.2017.11.001>
120. Wilson, P. H., Rogers, J. M., Vogel, K., Steenbergen, B., McGuckian, T. B., & Duckworth, J. (2021). Home-based (virtual) rehabilitation improves motor and cognitive function for stroke patients: a randomized controlled trial of the Elements (EDNA-22) system. J Neuroeng Rehabil, 18(1), 165. <https://doi.org/10.1186/s12984-021-00956-7>
121. Yague, S., De Las Heras, M. V., Pedro, J., Martinez-Yelamos, A., Cardona, P., Quesada, H., Lara, B., Kumru, H., Garcia, B., Montero, J., & Valls-Sole, J. (2018). Effects of bihemispheric tDCS combined with radial nerve stimulation in acute stroke patients. Clinical Neurophysiology, 129(Supplement 1), e48-e49. <https://doi.org/https://dx.doi.org/10.1016/j.clinph.2018.04.121> (31st International Congress of Clinical Neurophysiology, ICCN of the IFCN. Washington, DC United States.)
122. Yazdani, M., Chitsaz, A., Zolaktaf, V., Saadatnia, M., Ghasemi, M., Nazari, F., Chitsaz, A., Suzuki, K., & Nobari, H. (2022). Can Early Neuromuscular Rehabilitation Protocol Improve Disability after a Hemiparetic Stroke? A Pilot Study. Brain Sci, 12(7). <https://doi.org/10.3390/brainsci12070816>
123. Yen, C.-C., Chen, H.-H., Lee, C.-H., & Lin, C.-H. (2023). Predictive value of motor-evoked potentials for motor recovery in patients with hemiparesis secondary to acute ischemic stroke. Annals of medicine, 55(1), 2225144. <https://doi.org/https://dx.doi.org/10.1080/07853890.2023.2225144>
124. Yu, C., Wang, W., Zhang, Y., Wang, Y., Hou, W., Liu, S., Gao, C., Wang, C., Mo, L., & Wu, J. (2017). The Effects of Modified Constraint-Induced Movement Therapy in Acute Subcortical Cerebral Infarction. Front Hum Neurosci, 11, 265. <https://doi.org/10.3389/fnhum.2017.00265>
125. Zhang, J., & Chang, Y. (2023). Alterations of static and dynamic functional network connectivity in acute ischemic brainstem stroke. Acta radiologica (Stockholm, Sweden : 1987), 64(4), 1623-1630. <https://doi.org/https://dx.doi.org/10.1177/02841851221127271>
126. Zhang, Z., Sun, X., Liu, X., Wang, L., & Zhu, R. (2022). Clinical features, etiology, and prognosis of hand knob stroke: a case series. BMC Neurol, 22(1), 331. <https://doi.org/10.1186/s12883-022-02858-0>
127. Zhang, S., Yu, Y., Xu, P., Shen, X., Fang, C., Wu, X., Qu, P., Wu, T., Wang, Q. M., Luo, X., & Hong, Y. (2023). Mechanical digit sensory stimulation: a randomized control trial on neurological and motor recovery in acute stroke. Front Neurosci, 17, 1134904. <https://doi.org/10.3389/fnins.2023.1134904>
128. Zhao, L., Liu, Z., Sun, Q., & Li, H. (2022). Effect of transcranial direct current stimulation combined with a smart hand joint training device on hand dysfunction in patients with early stroke. Folia neuropathologica, 60(2), 177-184. https://doi.org/https://dx.doi.org/10.5114/fn.2022.117534
129. Wang, H.-C., Chou, W., You, Y.-L., Wang, Y.-L., Hsu, M., Yang, C.-C., Yen, C.-W., Gou, L.-Y., & GUO, L.-Y. (2024). Effects of Thermal Stimulation and Transcutaneous Electrical Nerve Stimulation on Sensory and Motor Function of Upper Extremity in Acute Stroke Survivors: A Randomized Controlled Pilot Study. Cureus, 16(6).
130. Veerbeek, J. M., Ruhe, H., Ottiger, B., Bohlhalter, S., Nyffeler, T., & Cazzoli, D. (2025). Impact of Neglect on the Relationship Between Upper Limb Motor Function and Upper Limb Performance in the (Hyper)acute Poststroke Phase. Neurorehabilitation and Neural Repair, 39(2), 138 <https://doi.org/https://dx.doi.org/10.1177/15459683241304329>
131. Vora, I., Huynh, B. P., Lin, D. J., & Kimberley, T. J. (2025). MEP status revisited: Potential value of the MEP trichotomy to distinguish arm motor behavior [Medical Treatment of Physical Illness 3363]. Brain Stimulation, 18(2), 262-264. <https://doi.org/https://dx.doi.org/10.1016/j.brs.2025.02.014>
132. Zich, C., Ward, N. S., Forss, N., Bestmann, S., Quinn, A. J., Karhunen, E., & Laaksonen, K. (2025). Post-stroke changes in brain structure and function can both influence acute upper limb function and subsequent recovery. NeuroImage: Clinical, 45, 103754. <https://doi.org/https://dx.doi.org/10.1016/j.nicl.2025.103754>

**Supplementary Results**

**Study Demographics for clinical trials**

The highest number of publications originated from China (*N* = 8) and the United States of America (*N* = 4, **SI Fig. 1a**). Sample sizes ranged from 16 to 610 participants (*M* = 76.0, *Med* = 42, *SD* = 112.1, *SE* = 20.8), with 24.1% (*N* = 7) of studies having 25 or fewer subjects (**SI Fig. 1b**). On average more male than female participants were included in these studies (Male: *M* = 43.9, *Med* = 22.0, *SD* = 66.9, *SE* = 12.4; Female: *M* = 32.1, *Med* = 23.0, *SD* = 46.0, *SE* = 8.5; *t (56)* = 2.6, *p* =0.014). The mean age across studies was 64.3 years (*Med* = 64.3, *SD* = 5.3, *SE* = 1.0, **SI Fig. 1c**). Amongst the studies providing an age range (37.9% [*N* = 11] of the studies) only 45.5% (*N* = 5) have a minimum age below 35 years (**SI Fig. 1c**).

***
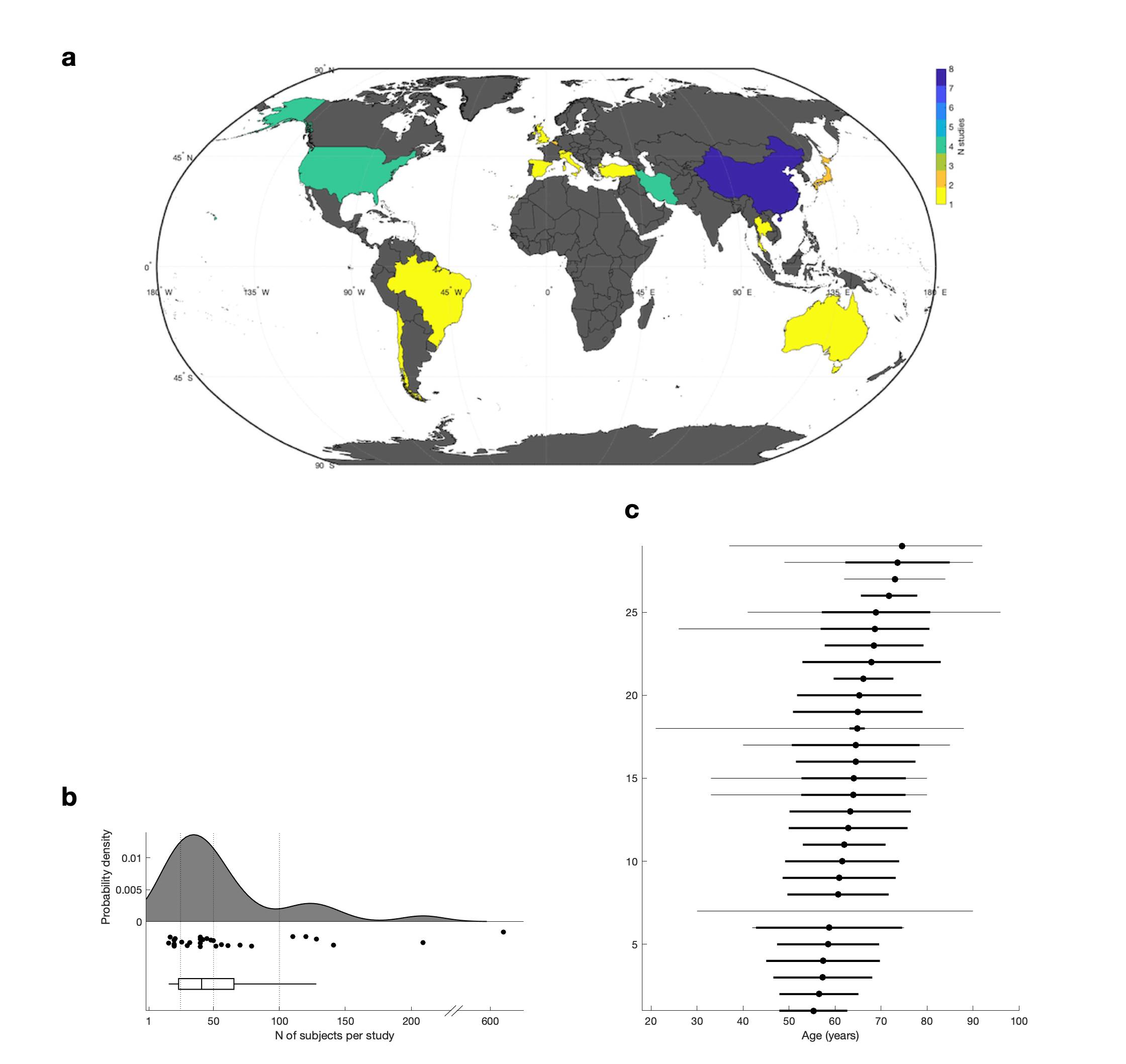
SI Fig. 1. Study Demographics for clinical trials***

*a) Geographical distribution of clinical trials.*

*b) Number of subjects per trial. Shown is the probability function (top), the corresponding individual datapoints (middle), and the boxplot (bottom). Horizontal line indicates N = 25, N = 50, and N = 100.*

*c) Age of subjects per trial. Each row represents one study in the scoping review, which are sorted based on the average age (i.e., mean or median, depending on data availability). Average age is shown as a block dot, variance (i.e., standard deviation) is shown as thick black line, and age range is shown as thin grey line.*

***
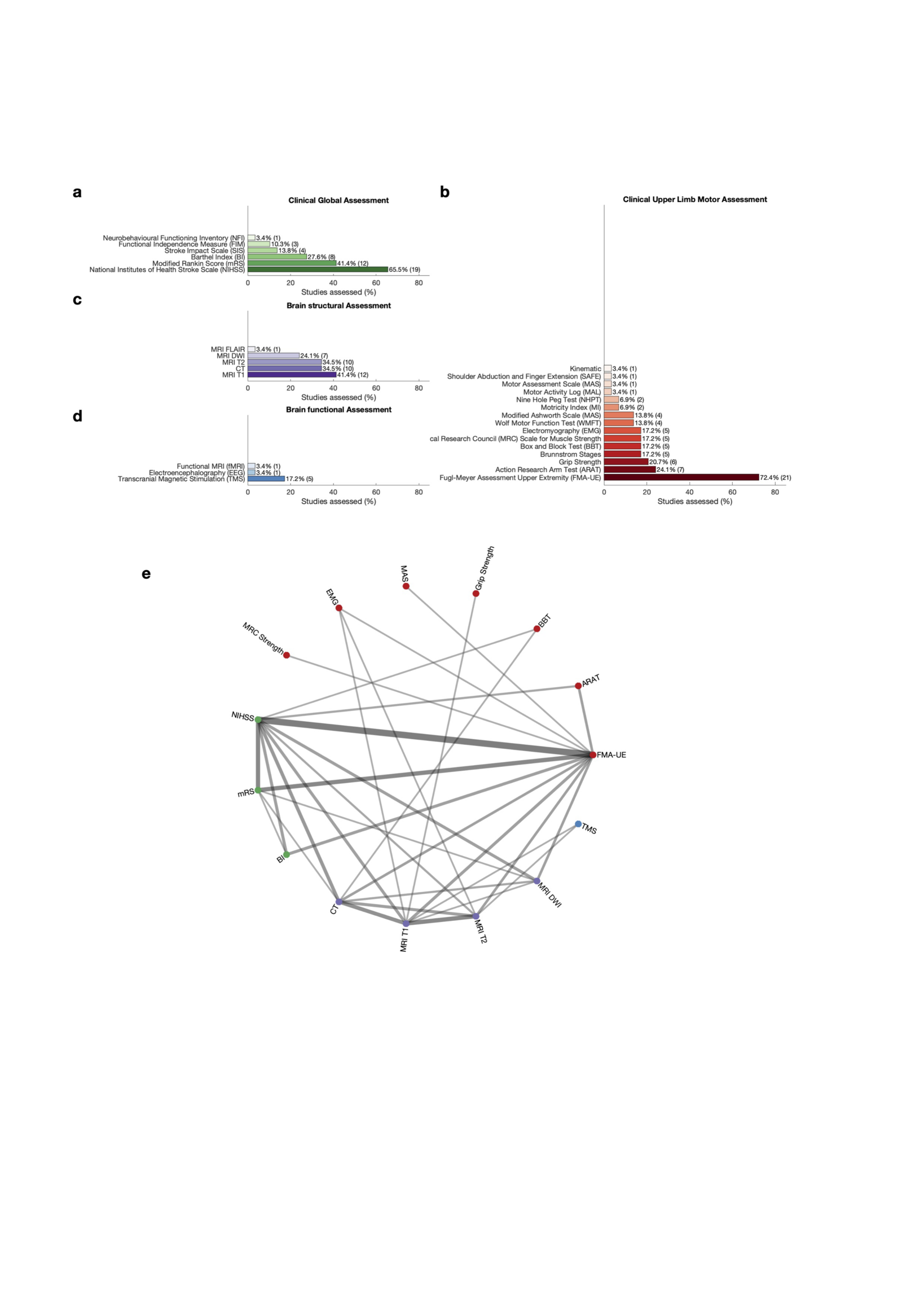
***

***SI Fig. 2. Percentage of Assessment categorised by Assessment type for clinical trials only***

*a) Clinical Global Assessment. Assessments are ranked and coloured by the frequency of which they are used.*

*b) Clinical upper limb Motor Assessment. Assessments are ranked and coloured by the frequency of which they are used.*

*c) Brain structural Assessment. Assessments are ranked and coloured by the frequency of which they are used.*

*d) Brain functional Assessment. Assessments are ranked and coloured by the frequency of which they are used.*

*e) Connectivity plot indicating which Assessments are obtained together in one study. The colour of each node corresponds to the assessment category; green for global clinical assessments, red for motor assessments, blue for measures of brain function, and purple for measures of brain structures. Colour and thickness of the edge between two nodes indicates the frequency of which two assessments are used together in one study. Connections that occur less than 3% (N < 5) are omitted from the plot.*
